# Long-term health status and trajectories of seriously injured patients: A population-based longitudinal study

**DOI:** 10.1371/journal.pmed.1002322

**Published:** 2017-07-05

**Authors:** Belinda J. Gabbe, Pam M. Simpson, Peter A. Cameron, Jennie Ponsford, Ronan A. Lyons, Alex Collie, Mark Fitzgerald, Rodney Judson, Warwick J. Teague, Sandra Braaf, Andrew Nunn, Shanthi Ameratunga, James E. Harrison

**Affiliations:** 1 School of Public Health and Preventive Medicine, Monash University, Melbourne, Victoria, Australia; 2 Farr Institute, Swansea University Medical School, Swansea University, Swansea, United Kingdom; 3 Emergency and Trauma Centre, The Alfred, Melbourne, Victoria, Australia; 4 Monash-Epworth Rehabilitation Research Centre, Melbourne, Victoria, Australia; 5 School of Psychological Sciences, Monash University, Melbourne, Victoria, Australia; 6 Insurance Work and Health Group, Faculty of Medicine Nursing and Health Sciences, Monash University, Melbourne, Victoria, Australia; 7 Trauma Service, The Alfred, Melbourne, Victoria, Australia; 8 Department of Surgery, Monash University, Melbourne, Victoria, Australia; 9 Trauma Service, Royal Melbourne Hospital, Parkville, Victoria, Australia; 10 Trauma Service, The Royal Children’s Hospital, Melbourne, Victoria, Australia; 11 Department of Paediatrics, University of Melbourne, Melbourne, Victoria, Australia; 12 Surgical Research Group, Murdoch Children’s Research Institute, Melbourne, Victoria, Australia; 13 Victorian Spinal Cord Service, Austin Health, Heidelberg, Victoria, Australia; 14 Section of Epidemiology and Biostatistics, School of Population Health, University of Auckland, Auckland, New Zealand; 15 Research Centre for Injury Studies, Flinders University, Adelaide, South Australia, Australia; Barts and the London School of Medicine & Dentistry Queen Mary University of London, UNITED KINGDOM

## Abstract

**Background:**

Improved understanding of the quality of survival of patients is crucial in evaluating trauma care, understanding recovery patterns and timeframes, and informing healthcare, social, and disability service provision. We aimed to describe the longer-term health status of seriously injured patients, identify predictors of outcome, and establish recovery trajectories by population characteristics.

**Methods and findings:**

A population-based, prospective cohort study using the Victorian State Trauma Registry (VSTR) was undertaken. We followed up 2,757 adult patients, injured between July 2011 and June 2012, through deaths registry linkage and telephone interview at 6-, 12-, 24-, and 36-months postinjury. The 3-level EuroQol 5 dimensions questionnaire (EQ-5D-3L) was collected, and mixed-effects regression modelling was used to identify predictors of outcome, and recovery trajectories, for the EQ-5D-3L items and summary score. Mean (SD) age of participants was 50.8 (21.6) years, and 72% were male. Twelve percent (*n* = 333) died during their hospital stay, 8.1% (*n* = 222) of patients died postdischarge, and 155 (7.0%) were known to have survived to 36-months postinjury but were lost to follow-up at all time points. The prevalence of reporting problems at 36-months postinjury was 37% for mobility, 21% for self-care, 47% for usual activities, 50% for pain/discomfort, and 41% for anxiety/depression. Continued improvement to 36-months postinjury was only present for the usual activities item; the adjusted relative risk (ARR) of reporting problems decreased from 6 to 12 (ARR 0.87, 95% CI: 0.83–0.90), 12 to 24 (ARR 0.94, 95% CI: 0.90–0.98), and 24 to 36 months (ARR 0.95, 95% CI: 0.95–0.99). The risk of reporting problems with pain or discomfort increased from 24- to 36-months postinjury (ARR 1.06, 95% CI: 1.01, 1.12). While loss to follow-up was low, there was responder bias with patients injured in intentional events, younger, and less seriously injured patients less likely to participate; therefore, these patient subgroups were underrepresented in the study findings.

**Conclusions:**

The prevalence of ongoing problems at 3-years postinjury is high, confirming that serious injury is frequently a chronic disorder. These findings have implications for trauma system design. Investment in interventions to reduce the longer-term impact of injuries is needed, and greater investment in primary prevention is needed.

## Abstract 2

In a population-based longitudinal study, Belinda Gabbe and colleagues report 3-year outcomes for seriously injured patients in Victoria, Australia.

## Author summary

**Why was this study done?:**

- Improvements in trauma care have improved the chances of surviving serious injury, requiring a shift in focus to better understanding how well people recover from injury and how long this takes.
- Longitudinal studies of the long-term health outcomes of seriously injured people are few. This study was undertaken to close this knowledge gap and provide valuable data necessary to inform trauma system design, injury rehabilitation programs, compensation schemes, and estimates of injury burden.

**What did the researchers do and find?:**

- We followed a cohort of 2,757 major trauma patients in Victoria, Australia at 6 months, 12 months, 24 months, and 36 months after injury to collect health outcomes using the 3-level EuroQol 5 dimensions questionnaire (EQ-5D-3L).
- We found that 20% of patients had died by 36-months postinjury. The proportion of survivors reporting persistent problems was high for each of the EQ-5D-3L items, although improvement was continuing at 36 months after injury for the usual activities item.
- After adjusting our analyses to account for possible confounding factors, we found that lower levels of education, claiming compensation for injury, and age were consistent predictors of reporting problems at follow-up. The nature of injuries sustained, gender, preinjury employment, and level of socioeconomic disadvantage were also important predictors of problems on many of the 5 EQ-5D-3L items.

**What do these findings mean?:**

- The prevalence of ongoing problems following serious injury was high at 36 months, though continued improvement was evident.
- Investment in interventions designed to prevent major trauma overall, and to reduce the impact of injury, is clearly needed.

## Introduction

The implementation of organised trauma systems has enabled considerable reductions in injury-related mortality in high-income countries [1–3]. With improving survival rates comes the potential for greater numbers of people living with long-term injury impacts, including reduced health status or health-related quality of life. An expert consensus group identified the capture of functional and quality-of-life outcomes following trauma as critically important for improving healthcare quality more than 2 decades ago [4]. Despite this, integration of these outcomes into trauma registries and system monitoring has been largely absent, and calls for their inclusion continue [5,6].

Improved understanding of the quality of survival is crucial in evaluating the quality of care provided to trauma patients. This information is important for understanding the patterns and timeframe of recovery and for best informing provision of healthcare, social, and disability services to those with ongoing issues following injury [6–8]. Establishing rates and patterns of recovery, and how these change over time, requires longitudinal data to inform what to measure, when, and for how long. To date, longitudinal studies of the health status of trauma patients have typically limited follow-up to 12- or 24-months postinjury [8–15]. Many have focused on less severely injured patients [10,11,13,14] and found little improvement beyond 9- to 12-months postinjury [10,12,14]. Previous studies of major trauma patients have shown continued improvement in function, return to work, and pain outcomes to 2 years after injury [8,9,16,17], and recent injury studies have shown variable trajectories of recovery in the first 2 years after injury [8,11]. However, studies extending beyond 2 years after injury are few and have involved very small cohorts [18,19], with 1 study showing improvement in physical health and function 10 years following injury when compared to 5 years in a cohort of 58 patients [18]. Our understanding of how long it takes to recover, and the factors that influence the degree and time course of recovery, after serious injury remains incomplete.

The aims of this population-based study were to: (i) describe the health status of seriously injured patients over a 3-year follow-up period, (ii) identify predictors of health status, and (iii) establish whether recovery trajectories differ by key patient and injury characteristics.

## Methods

The Victorian State Trauma Registry (VSTR) and the REcovery after Serious Trauma—Outcomes, Resource use, and patient Experiences (RESTORE) project have been approved by the Human Research Ethics Committee of each participating hospital and Monash University.

### Setting and participants

The state of Victoria has a population 5.8 million and represents approximately 25% of the Australian population. An integrated trauma system was established in 2000 and is monitored using the VSTR, which captures data about all hospitalised major trauma cases. Major trauma is defined as any of the following: death following injury, an Injury Severity Score >12, urgent surgery, or admission to intensive care for >24h [20]. The registry data are regularly linked with the Victorian deaths registry to identify postdischarge deaths. Survivors to hospital discharge are routinely followed-up by telephone at 6, 12, and 24 months after injury to collect data about return to work, function, pain, and health status. The RESTORE study is extending the timeframe for follow-up to 36-, 48-, and 60-months postinjury for all patients with a date of injury from July 2011 to June 2012 [21]. Adult (aged 18 years and over) patients from the RESTORE project were included in this study.

Eligible patients were provided with a letter and brochure about the registry, explaining what is collected, how the data are used, and how to have their details removed. At each telephone interview, verbal consent to complete the interview was obtained.

While Australia’s publicly funded healthcare system (Medicare) provides healthcare coverage for all Australian citizens and permanent residents, 57% of the adult population purchase private health insurance. Additionally, Victoria has no-fault third party insurers for road (Transport Accident Commission [TAC]) and work-related (WorkSafe Victoria) injury, which provide compensation for treatment, rehabilitation, income replacement, and long-term support services.

### Procedures

The protocol for the RESTORE study is described elsewhere but summarised here [21]. Survivors to hospital discharge were telephoned at 6-, 12-, 24-, and 36-months postinjury using a standardised interview, which included validated patient-reported outcome measures. The collection of outcomes is also planned at 48- and 60-months postinjury. The health-status measure used was the 3-level EuroQol 5 dimensions questionnaire (EQ-5D-3L), comprising 5 items, including mobility, usual activities (e.g., work, study, housework, family, or leisure activities), self-care, pain or discomfort, and anxiety or depression [22]. For each item, the level of problems experienced is measured on a 3-point scale: no problems, some problems, severe problems. Age- and gender-specific population weights (tariffs) are applied to the item responses to generate preference weights, resulting in a utility score ranging from −0.594 to 1, in which 0 represents a health state equivalent to death, 1 represents perfect health, and a score <0 represents a health state considered worse than death. The United Kingdom value sets (or tariffs) were used for this study to calculate EQ-5D-3L preference weights, as these are most commonly used [23].

Demographic factors, injury event, injury type and severity, and other relevant factors were extracted from the registry and RESTORE for analysis. The registry receives the 10th Revision of the International Classification of Diseases—Australian Modification (ICD-10-AM) codes for each trauma patient. The ICD-10-AM coding is a requirement for all hospital admissions in Australia. The Charlson Comorbidity Index (CCI) and presence of preexisting mental health, drug, or alcohol conditions were mapped from the ICD-10-AM codes for each patient using published algorithms [24,25]. The CCI weight was used for analysis: 0 representing no CCI conditions, 1 representing at least 1 CCI condition with a weight of 1, and 2+ representing patients with at least 1 CCI condition with a weight of 2 or greater [24]. Socioeconomic status was obtained by applying the Index of Relative Socioeconomic Advantage and Disadvantage (IRSAD) to the patient’s postcode of residence to obtain quintiles of socioeconomic advantage ranging from 1 (most disadvantaged) to 5 (most advantage). Geographic remoteness was assessed by applying the Accessibility/Remoteness Index of Australia (ARIA), which classifies the patient’s postcode of residence into 1 of 5 categories: major city, inner regional, outer regional, remote, or very remote. These categories were collapsed into 2 categories, major city and all others combined, as the number of cases in regional and remote areas was low. Abbreviated Injury Scale 2005 (2008 revision) diagnosis codes were used to categorise each patient’s injuries into 1 of 7 nature-of-injury groups. These groups represent the 6 most common nature-of-injury groups and 1 residual group representing patients with burns or multiple injuries but without serious neurotrauma. The cause of injury categories was collapsed for analysis into the 6 most common causes of injury (motor vehicle occupant, motorcyclist, pedestrian or pedal cyclist, low fall, high fall, and struck by or collision with an object or person) and 1 residual category. Age was categorised into 7 groups for ease of interpretation of the findings and because the relationship with outcome was not linear. Self-reported preinjury disability was determined using a validated question asking the patient’s level of disability in the week prior to injury with options of none, mild, moderate, marked, or severe [26]. The marked and severe categories were combined for analysis due to the low number of patients reporting preinjury disability in these categories.

### Data analysis

Frequencies, percentages, and the 95% confidence intervals (CI) of the percentages were used for categorical variables, and mean and SD were used for continuous variables. Patients were considered lost to follow-up if the EQ-5D-3L was missing at every follow-up time point (6-, 12-, 24-, and 36-months postinjury), and the patient had not died since hospital discharge.

Predictors of outcomes were assessed using mixed effects regression models [27]. All models included covariates previously identified as predictors of outcome in published studies and were adjusted for self-reported levels of preinjury disability. Both the CCI and self-reported preinjury level of disability were included in the model, as these represent separate constructs of preinjury health and function. Linear regression was used for the EQ-5D-3L preference or utility score, based on the age- and gender-specific population weights (tariffs), and a modified Poisson model was used for each of the 5 items, in which responses were dichotomised into no problems versus some/severe problems. Whilst the protocol paper was closely followed, a modified Poisson model was used to allow the estimation of relative risks (RRs) rather than odds ratios for the binary outcomes, as RRs are the preferred estimate for prospective studies [28]. The modified Poisson method has been shown to provide consistent results with a log-binomial model in which average risk levels are low to moderate, as observed in our data [29]. Similarly, the relative efficiency of the log-Poisson model is high compared to the log-binomial model at the average risk levels observed in our study [29]. Adjusted mean differences and 95% CI were calculated for linear models. Adjusted RR and 95% CI were calculated for binary outcome models. Differences in change over time in the prevalence of each outcome between patient subgroups (e.g., males versus females) were assessed using an interaction term between each covariate and time postinjury, with the adjusted mean difference and RR representing the difference in mean scores, or risk of improvement in outcome, in that group relative to the previous time point, respectively. These tests for interactions were performed to ascertain whether outcome trajectories differed between categories of the covariates—for example, whether the change in prevalence of the outcome differed over time for men compared to women.

Data were missing for some covariates: compensable status (*n* = 17, 0.7%); injury intent (*n* = 21, 0.9%); presence of a preexisting mental health, drug, or alcohol condition (*n* = 65, 2.9%); geographic remoteness and socioeconomic status (*n* = 65, 2.9%); preinjury work status (*n* = 107, 4.7%); preinjury level of disability (*n* = 115, 5.1%); and highest level of education (*n* = 315, 13.9%). Missing covariates were imputed using multiple imputation by chained equations. All covariates, and each outcome from the study, were used in the process to impute the missing covariate data. However, the data points with missing outcomes were not used in the modelling procedures [30]. Twenty datasets were produced, which were then combined using Rubin’s rules, a multiple-imputation algorithm [31]. A *P* value less than 0.05 was considered significant, and all analyses were performed using Stata Version 13.

## Results

### Cohort description and follow-up

**Fig 1:**
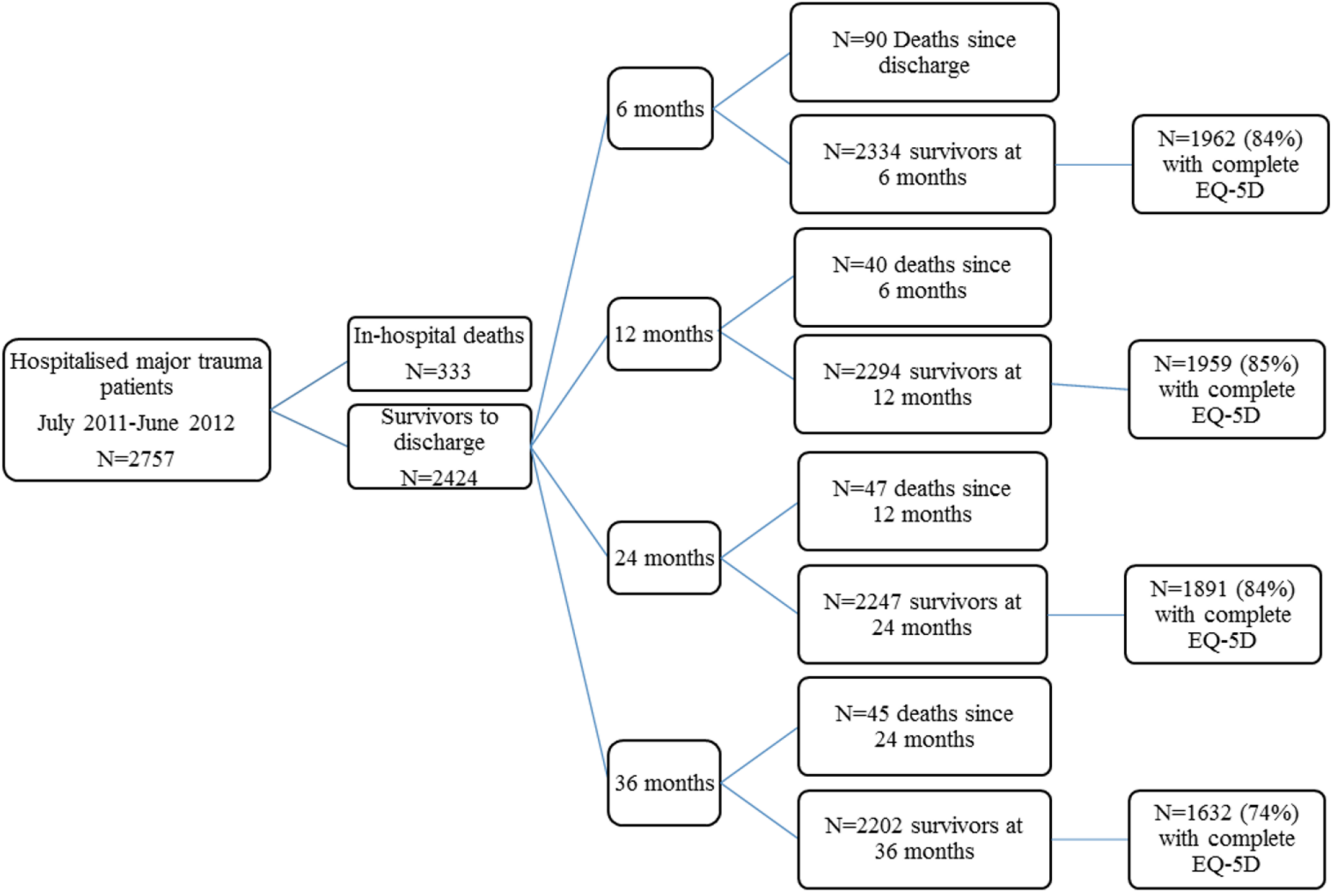
Flow of participants through the study.

There were 2,757 hospitalised major trauma patients in Victoria with a date of injury from July 2011 to June 2012; 12.1% (*n* = 333) died during their hospital stay, and a further 222 (8.1%) patients died postdischarge (Fig 1). The follow-up rates were 84% at 6-months, 85% at 12-months, 84% at 24-months and 74% at 36-months postinjury (Fig 1). The mean (SD) age of participants was 50.8 (21.6) years, and 72% were male. Ninety-two percent of cases were blunt trauma, with falls and road transport being the most common causes of injury. Over the study timeframe, 155 (7.0%) survivors to 36-months postinjury did not complete the 3-level EuroQol 5 dimensions questionnaire (EQ-5D-3L) at any time point and were considered lost to follow-up. Patients lost to follow-up were younger; less severely injured; more commonly injured through self-harm or assault; more commonly had a penetrating trauma type; had a higher prevalence of mental health, drug, or alcohol conditions; and more commonly lived in major cities (Table 1).

**Table 1:** Profile of patients successfully followed up and patients lost to follow-up.

**Population characteristic**		**Followed up** **(*N* = 2,269)**	**Complete loss to follow-up** **(*N* = 155)**	***P* value**
**Age group**	*N* (%: 95% CI)			<0.001
	18–24 years	303 (13.4%: 12.0% to 14.8%)	36 (23.2%: 17.2% to 30.5%)	
	25–34 years	321 (14.2%: 12.8% to 15.6%)	42 (27.1%: 20.7% to 34.7%)	
	35–44 years	315 (13.9%: 12.5% to 15.4%)	25 (16.1%: 11.1% to 22.8%)	
	45–54 years	315 (13.9%: 12.5% to 15.4%)	25 (16.1%: 11.1% to 22.8%)	
	55–64 years	325 (14.3%: 12.9% to 15.8%)	15 (9.7%: 5.9% to 15.5%)	
	65–74 years	255 (11.2%: 10.0% to 12.6%)	4 (2.6%: 0.9% to 6.7%)	
	75+ years	435 (19.2%: 17.6% to 20.8%)	8 (5.2%: 2.6% to 10.0%)	
**Gender**	*N* (%: 95% CI)			0.07
	Male	1,615 (71.2%: 69.3% to 73.0%)	121 (78.1%: 70.8% to 83.9%)	
	Female	654 (28.8%: 27.0% to 30.7%)	34 (21.9%: 16.1% to 29.2%)	
**Compensable status^a^**	*N* (%: 95% CI)			0.34
	Not compensable	1,294 (57.5%: 55.4% to 59.5%)	94 (61.4%: 53.4% to 68.8%)	
	Compensable	958 (42.5%: 40.5% to 44.6%))	59 (38.6%: 31.2% to 46.5%)	
**Charlson Comorbidity Index**	*N* (%: 95% CI)			0.09
	None	1,474 (65.0%: 63.0% to 66.9%)	99 (63.9%: 56.0% to 71.1%)	
	1	594 (26.2%: 24.4% to 28.0%)	49 (31.6%: 24.8% to 39.4%)	
	2+	201 (8.8%; 7.8% to 10.1%)	7 (4.5%: 2.2% to 9.2%)	
**Preexisting mental health/drug/alcohol condition^b^**	*N* (%: 95% CI)			<0.001
	No	1,618 (73.4%: 71.5% to 75.2%)	91 (60.3%: 52.2% to 67.8%)	
	Yes	586 (26.6%: 24.8% to 28.5%)	60 (39.7%: 32.2% to 47.8%)	
**Region of residence^c^**	*N* (%: 95% CI)			0.04
	Major cities	1,577 (71.6%: 69.6% to 73.4%)	114 (79.7%: 72.3% to 85.5%)	
	Regional or remote	627 (28.4%: 26.6% to 30.4%)	29 (20.3%: 14.5% to 27.7%)	
**Socioeconomic status (IRSAD)^c^**	*N* (%: 95% CI)			0.12
	1 (Most disadvantaged)	283 (12.8%: 11.5% to 14.3%)	25 (17.5%: 12.1% to 24.6%)	
	2	288 (13.1%: 11.7% to 14.5%)	14 (9.8%: 5.9% to 15.9%)	
	3	406 (18.4%: 16.9% to 20.1%)	33 (23.1%: 16.9% to 30.7%)	
	4	634 (28.8%: 26.9% to 30.7%)	31 (21.7%: 15.7% to 29.2%)	
	5 (Least disadvantaged)	593 (26.9%: 25.1% to 28.8%)	40 (28.0%: 21.2% to 35.9%)	
**Type of injury**	*N* (%: 95% CI)			<0.001
	Blunt	2,112 (93.1%: 92.0% to 94.1%)	127 (81.9%: 75.0% to 87.2%)	
	Penetrating	82 (3.6%: 2.9% to 4.5%)	21 (13.6%: 9.0% to 19.9%)	
	Burn	59 (2.6%: 2.0% to 3.3%)	4 (2.6%: 0.9% to 6.7%)	
	Asphyxiation/drowning	16 (0.7%: 0.4% to 1.1%)	3 (1.9%: 0.6% to 5.9%)	
**Cause of injury**	*N* (%: 95% CI)			<0.001
	Motor vehicle	537 (23.7%: 22.0% to 25.5%)	30 (19.3%: 13.9% to 26.4%)	
	Motorcycle	229 (10.1%: 8.9% to 11.4%)	19 (12.3%: 7.9% to 18.4%)	
	Pedal cyclist/pedestrian	247 (10.9%: 9.7% to 12.2%)	10 (6.4%: 3.5% to 11.6%)	
	Low fall (from standing or <1m)	526 (23.2%: 21.5% to 25.0%)	20 (12.9%: 8.5% to 19.2%)	
	High fall (>1m)	282 (12.4%: 11.1% to 13.9%)	19 (12.3%: 7.9% to 18.4%)	
	Struck by/collision	192 (8.5%: 7.4% to 9.7%)	27 (17.4%: 12.2% to 24.2%)	
	Other	256 (11.3%: 10.0% to 12.7%)	30 (19.4%: 13.9% to 26.4%)	
**Injury intent^d^**	*N* (%: 95% CI)			<0.001
	Unintentional	2,035 (90.5%: 89.2% to 91.7%)	107 (71.8%: 64.0% to 78.5%)	
	Intentional	213 (9.5%: 8.3% to 10.8%)	42 (28.2%: 21.5% to 36.0%)	
**Injury Severity Score**	*N* (%: 95% CI)			0.04
	<14	505 (22.3%: 20.6% to 24.0%)	49 (31.6%: 24.8% to 39.4%)	
	14–16	462 (20.4%: 18.8% to 22.1%)	32 (20.7%: 15.0% to 27.8%)	
	17–21	580 (25.6%: 23.8% to 27.4%)	37 (23.9%: 17.8% to 31.2%)	
	22+	720 (31.8%: 29.9% to 33.7%)	37 (23.9%: 17.8% to 31.2%)	
**Nature of injury**	*N* (%: 95% CI)			0.001
	Isolated head injury	341 (15.0%: 13.6% to 16.6%)	16 (10.3%: 6.4% to 16.2%)	
	Head and other injuries	517 (22.8%: 21.1% to 24.6%)	30 (19.3%: 13.9% to 26.4%)	
	Spinal cord injury	66 (2.9%: 2.3% to 3.7%)	1 (0.7%: 0.1% to 4.5%)	
	Orthopaedic injuries only	217 (9.6%: 8.4% to 10.8%)	19 (12.3%: 7.9% to 18.4%)	
	Chest/abdominal injuries alone	145 (6.4%: 5.5% to 7.5%)	20 (12.9%: 8.5% to 19.2%)	
	Chest/abdominal and other injuries	636 (28.0%: 26.2% to 29.9%)	34 (21.9%: 16.1% to 29.2%)	
	Other multitrauma and burns	347 (15.3%: 13.9% to 16.8%)	35 (22.6%: 16.7% to 29.9%)	
**Level of definitive care**	*N* (%: 95% CI)			0.55
	Major trauma service	1,860 (82.0%: 80.3% to 83.5%)	130 (83.9%: 77.2% to 88.9%)	
	Nonmajor trauma service	409 (18.0%: 16.5% to 19.7%)	25 (16.1%: 11.1% to 22.8%)	

^a^ *n* = 17 missing

^b^ *n* = 69 missing

^c^ *n* = 77 missing

^d^ *n* = 27 missing, intentional includes intentional self-harm and interpersonal violence.

CI, confidence intervals; IRSAD, Index of Relative Socioeconomic Advantage and Disadvantage.

### Overall change in recovery

**Fig 2:**
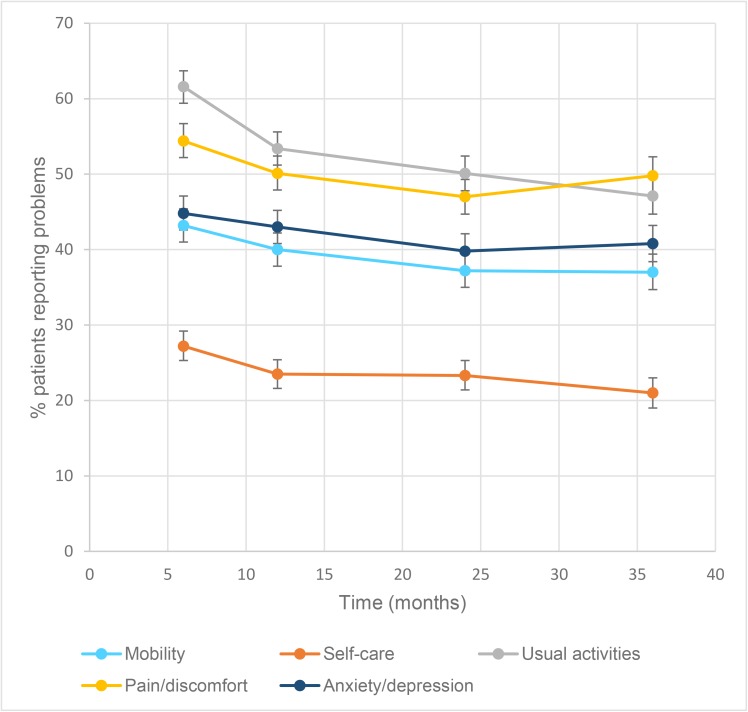
Percentage (95% confidence intervals [CI]) of patients reporting problems on each 3-level EuroQol 5 dimensions questionnaire (EQ-5D-3L) item at each time point postinjury.

The pattern of change in the prevalence of patients reporting problems at each time point postinjury differed by EQ-5D-3L item (Fig 2 and Table 2). The usual activities item was the only outcome where improvement (compared with the previous time point) was observed at every time point after injury. There was a 13% (ARR 0.87, 95% CI 0.83–0.90) reduction in the prevalence and risk of reporting problems with usual activities at 12 months compared to 6 months, a 6% (ARR 0.94, 95% CI 0.90–0.98) reduction from 12 months to 24 months, and a 5% (ARR 0.95, 95% CI: 0.90, 0.99) reduction from 24 months to 36 months. The risk of reporting problems with self-care decreased by 14% (ARR 0.86, 95% CI 0.80–0.93) from 6- to 12-months postinjury but showed no improvement after 12-months postinjury, while the ARR of reporting mobility problems declined 7% (ARR 0.93, 95% CI 0.89–0.97) from 6 to 12 months after injury and a further 6% (ARR 0.94, 95% CI 0.90–0.99) from 12 to 24 months. Pain or discomfort outcomes improved 8% (ARR 0.92, 95% CI 0.88–0.96) from 6 to 12 months and to 6% (ARR 0.94, 95% CI 0.89–0.98) from 12 to 24 months, but there was a 6% (ARR 1.06, 95% CI 1.01–1.12) increase in the ARR of reporting problems with pain or discomfort from 24- to 36-months postinjury (Table 2). Improvement in anxiety or depression outcomes was only observed between 12- and 24-months postinjury. Overall health status, as measured by the EQ-5D-3L summary score, showed significant improvement from 6- to 12- and 12- to 24-months postinjury, before declining from 24-months to 36-months postinjury, driven by the poorer pain outcomes at 36 months (Table 2). The mean (SD) summary score at 6-months postinjury was 0.67 (0.31), 0.68 (0.32) at 12-months postinjury, 0.71 (0.31) at 24-months postinjury, and 0.70 (0.32) at 36-months postinjury.

**Table 2:** Longitudinal analysis of change in EQ-5D-3L over time adjusted for key covariates.

	12 months	24 months	36 months
	*ARR compared to 6 months (95% CI)	*ARR compared to 12 months (95% CI)	*ARR compared to 24 months (95% CI)
Mobility	0.93 (0.89, 0.97)	0.94 (0.90, 0.99)	1.02 (0.97, 1.08)
Self-care	0.86 (0.80, 0.93)	1.01 (0.94, 1.09)	0.94 (0.86, 1.02)
Usual activities	0.87 (0.83, 0.90)	0.94 (0.90, 0.98)	0.95 (0.90, 0.99)
Pain/discomfort	0.92 (0.88, 0.96)	0.94 (0.89, 0.98)	1.06 (1.01, 1.12)
Anxiety/depression	0.95 (0.91, 1.00)	0.93 (0.88, 0.99)	1.03 (0.97, 1.10)
	Adjusted mean difference compared to 6 months (95% CI)	Adjusted mean difference compared to 12 months (95% CI)	Adjusted mean difference compared to 24 months (95% CI)
EQ-5D-3L summary score	0.02 (0.01, 0.03)	0.02 (0.01, 0.03)	−0.02 (−0.03, −0.01)

*Adjusted for age, gender, socioeconomic status (SES) as measured by the IRSAD, geographic remoteness, nature of injury, level of education, CCI, preinjury disability, cause and intent of injury, preexisting mental health, drug and alcohol conditions, highest level of education, preinjury work status, and whether managed at a major trauma service).

ARR, adjusted relative risk; CI, confidence intervals; CCI, Charlson Comorbidity Index; EQ-5D-3L, 3-level EuroQol 5 dimensions questionnaire; IRSAD, Index of Relative Socioeconomic Advantage and Disadvantage; SES, socioeconomic status.

### Predictors of outcome and differences in rates of recovery within covariates

**Fig 3:**
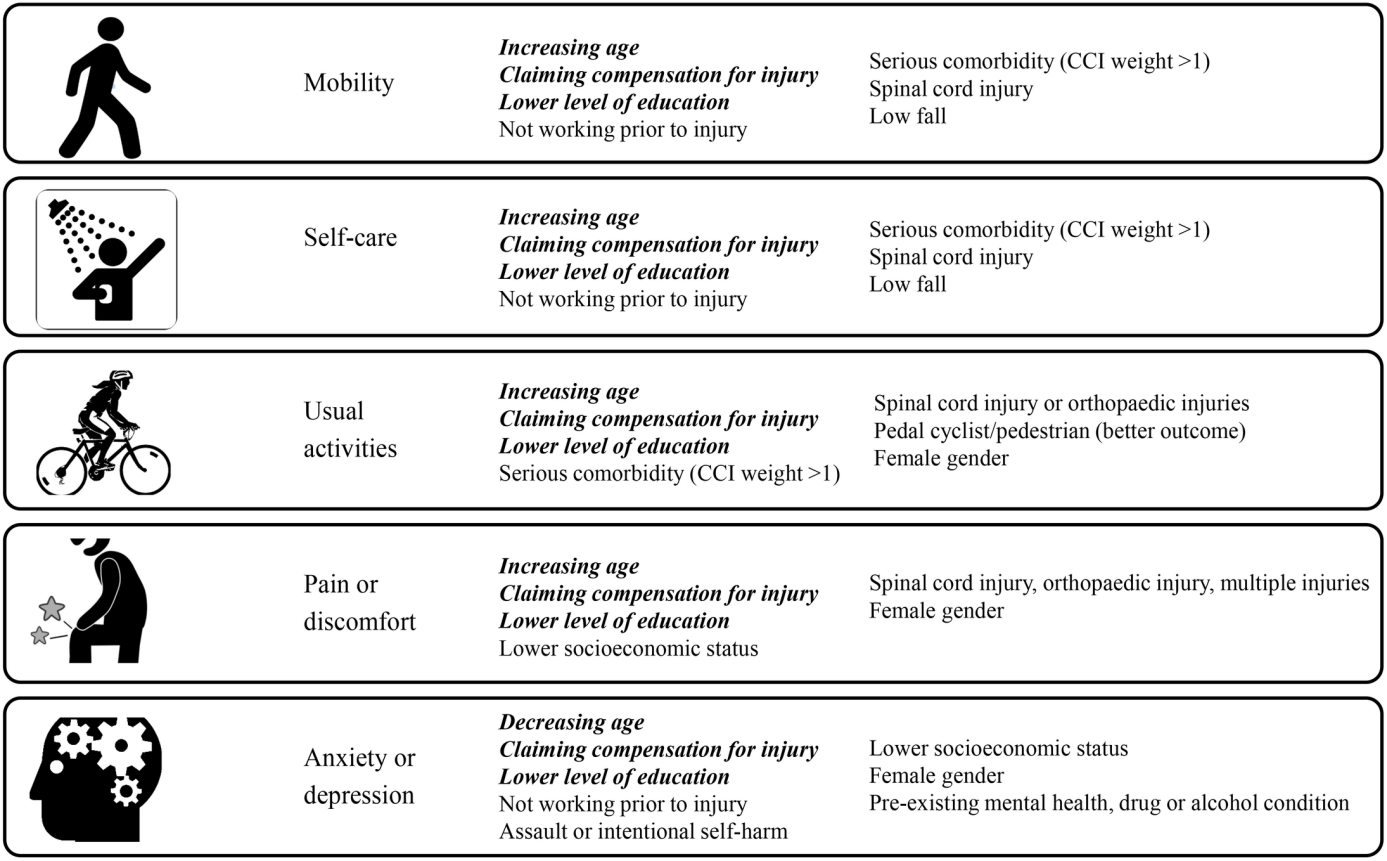
Summary of the multivariable modelling: Covariates predictive of reporting problems on each 3-level EuroQol 5 dimensions questionnaire (EQ-5D-3L) item at follow-up. (Factors predictive of all outcomes shown in bold and italics).

Fig 3 provides a summary of the key findings from the multivariable mixed-effects regression models investigating the predictors of reporting problems for each EQ-5D-3L item. The frequency and percentage of patients experiencing problems on each item, and the full results from the models, including the ARR and 95% CI are detailed in the supplementary material (S1 Table to S5 Table). Significant differences in change over time in the prevalence of each outcome between patient subgroups are noted in each section, and the full tables of these analyses are available on request from the authors.

#### Mobility

After adjusting for other factors and compared to 18 to 24-year-old patients, older patients experienced an increased risk of mobility problems; 25% (ARR 1.25, 95% CI 1.01–1.54) for 25–34 year olds, 28% (ARR 1.28, 95% CI 1.03–1.58) for 35–44 year olds, 48% (ARR 1.48, 95% CI 1.21–1.82) for 45–54 year olds, 43% (ARR 1.43, 95% CI 1.16–1.76) for 55–64 year olds, and 54% (ARR 1.54, 95% CI 1.25–1.91) for 65–74 year olds. The risk was more than 2-fold higher (ARR 2.05, 95% CI 1.66–2.53) in the group aged 75 years or older (S1 Table). Patients with compensable injury claims demonstrated a 92% (ARR 1.92, 95% CI 1.64–2.24) greater risk of reporting problems with mobility at follow-up, while patients working prior to injury experienced a 23% (ARR 0.77, 95% CI 0.68–0.87) lower risk of mobility problems (Fig 3 and S1 Table). A preexisting condition with a CCI weighting of 2 or greater was associated with a 21% (ARR 1.21, 95% CI 1.06–1.39) increased risk of mobility problems at follow-up. Compared with injured motor vehicle occupants, low falls patients were at a 38% (ARR 1.38, 95% CI 1.13–1.70) greater risk of mobility problems. Compared to patients with a university level education, patients with lower levels of education demonstrated a 28% greater risk of mobility problems (ARR 1.28, 95% CI 1.09–1.51) for diploma/certificate level and 29% (ARR 1.29, 95% CI 1.10–1.52) for patients not completing high school (S1 Table). Compared to patients sustaining isolated head injuries, spinal cord injured patients experienced a 2.6-fold (ARR 2.63, 95% CI 2.06–3.35) greater risk of mobility issues, while patients with only chest and abdominal injuries experienced a 35% (ARR 0.65, 95% CI 0.52–0.81) lower risk of mobility issues (Fig 3 and S1 Table). Age and working prior to injury showed a significant interaction with time postinjury, indicating different trajectories of recovery across the age groups and according to preinjury work status. For age, recovery trajectories differed, but there was no consistent pattern between the age groups. Patients who were working prior to injury showed improvement in mobility at each time point to 24 months, while patients not working prior to injury showed no improvement after 6-months postinjury.

#### Self-care

Increasing age, presence of preexisting conditions, lower education, a low-fall mechanism of injury, spinal cord injury, and claiming compensation were all associated with a higher risk of reporting problems with self-care at follow-up (Fig 3 and S2 Table). Compared to patients aged 18 to 24 years of age, the risk of reporting self-care problems increased 43% (ARR 1.43, 95% CI 1.05–1.96) for those aged 25–34 years, 50% (ARR 1.50, 95% CI 1.09–2.06) for 35–44 years, 93% (ARR 1.93, 95% CI 1.42–2.63) for 45–54 years, 79% (ARR 1.79, 95% CI 1.31–2.45) for 55–64 years, and 94% (ARR 1.94, 95% CI 1.39–2.70) for 65–74 years. This increased to a near 3-fold (ARR 2.75, 95% CI 1.99–3.80) risk of self-care problems in patients aged 75 years or older (S2 Table). Claiming compensation for injury was associated with a 97% (ARR 1.97, 95% CI: 1.57–2.47) increase in the risk of reporting self-care problems, while levels of education lower than a university degree also increased the risk of reporting self-care problems by 43% (ARR 1.43, 95% CI 1.13–1.82) for patients with a diploma or certificate and by 45% (ARR 1.45, 95% CI 1.14–1.84) for patients who did not finish high school (S2 Table). Patients working prior to injury experienced a 22% (ARR 0.78, 95% CI 0.65–0.93) lower risk of problems with self-care, while people sustaining only chest or abdominal injuries had a 34% (ARR 0.66, 95% CI 0.49, 0.88) lower risk of self-care problems at follow-up than patients with isolated head injuries. Compared to motor vehicle occupants, those injured in high falls showed a 33% (ARR 0.67, 95% CI 0.49–0.91) lower risk of reporting problems with self-care, while victims of low falls demonstrated a 57% (ARR 1.57, 95% CI 1.17–2.11) greater risk of self-care problems (Fig 3 and S2 Table). A preexisting condition with a CCI weight of 2 or greater was associated with an increased the risk of reporting self-care problems by 46% (ARR 1.46, 95% CI 1.20–1.77). The change over time in the prevalence of self-care problems differed by age group, with younger patients showing improvement at each time point on this item. Consistent with the mobility item, patients who were working prior to injury also showed improvement in self-care at each time point to 24-months postinjury, while patients not working prior to injury showed no improvement after 6-months postinjury.

#### Usual activities

After adjusting for other factors, older patients, women, compensable patients, patients with preinjury disability, spinal cord injury cases, and those claiming compensation for injury demonstrated higher risk of reporting problems with usual activities (Fig 3 and S3 Table). The ARR of reporting problems with usual activities was 11% (ARR 1.11, 95% CI 1.04–1.18) higher for women compared to men, 55% (ARR 1.55, 95% CI 1.38–1.73) higher for compensable patients compared to noncompensable patients, and 14% (ARR 1.14, 95% CI 1.03–1.25) higher for patients with a preexisting condition with a CCI weight of 2 or greater (S3 Table). The risk of reporting problems with usual activities increased with age. The risk increased by 20% (ARR 1.20, 95% CI 1.04–1.38) for the 25–34 year age group, 30% (ARR 1.30, 95% CI 1.13–1.49) for the 35–44 years group, 37% (ARR 1.37, 95% CI 1.19–1.57) for the 45–54 years group, 47% (ARR 1.47, 95% CI 1.28–1.68) for the 55–64 years group, 40% (ARR 1.40, 95% CI 1.20–1.63) for the 65–74 years group, and 59% (ARR 1.59, 95% CI 1.37–1.85) for the group 75 years of age and over (S3 Table). Compared to motor vehicle occupants, the risk of reporting problems on the usual activities item of the EQ-5D-3L was 15% (ARR 0.85, 95% CI 0.76–0.95) lower for pedal cyclists and pedestrians. The risk of problems with this item increased by 17% (ARR 1.17, 95% CI 1.02–1.35) for patients who finished high school, 25% (ARR 1.25, 95% CI 1.12–1.39) for patients with a diploma or certificate, and 28% (ARR 1.28, 95% CI 1.15–1.43) for patients who did not finish high school, when compared to patients with a university degree (S3 Table). The risk of reporting problems with usual activities was 70% (ARR 1.70, 95% CI 1.46–1.98) higher for spinal cord injured patients and 12% (ARR 1.12, 95% CI 1.00, 1.26) higher for patients with orthopaedic injuries when compared to patients with isolated head injuries (S3 Table). Patients who were working prior to injury showed improvement on this outcome to 24-months postinjury, while only patients aged 18 to 24 years showed significant improvement in self-care from 24- to 36-months postinjury.

#### Pain or discomfort

Women, older patients, compensable patients, and patients with spinal cord and orthopaedic injuries experienced greater risk of reporting problems with pain or discomfort at follow-up (Fig 3 and S4 Table). Compared to the most disadvantaged quintile, all other quintiles demonstrated a reduced risk of reporting problems with pain or discomfort—10% (ARR 0.90, 95% CI 0.80–1.00) for quintile 2, 10% (ARR 0.90, 95% CI 0.81–0.99) for quintile 3, 16% (ARR 0.84, 95% CI 0.77–0.93) for quintile 4, and 19% (ARR 0.81, 95% CI 0.73, 0.90) for quintile 5 (S4 Table). Compared with university-educated patients, lower education levels were associated with an increased risk of pain or discomfort—20% (ARR 1.20, 95% CI 1.05–1.37) for patients who completed high school, 27% (ARR 1.27, 95% CI 1.14–1.41) for patients with a diploma or certificate, and 26% (ARR 1.26, 95% CI 1.13–1.41) for patients who did not complete high school (Fig 3 and S4 Table). The risk of reporting pain or discomfort was 52% (ARR 1.52, 95% CI 1.37–1.69) higher for compensable patients compared to noncompensable patients, while women experienced a 17% (ARR 1.17, 95% CI 1.09–1.26) increased risk of pain or discomfort relative to men (S4 Table). Compared to patients with an isolated head injury, the presence of injuries to multiple body regions increased the risk of reporting pain or discomfort—21% (ARR 1.21, 95% CI 1.06–1.39) for patients with head and other injuries, 35% (ARR 1.35, 95% CI 1.20–1.57) for patients with chest, abdominal and other (nonneurotrauma) injuries, and 30% (ARR 1.30, 95% CI 1.12–1.49) for other multi-trauma patients and burns patients. Spinal cord injured patients and those with only orthopaedic injuries demonstrated a 92% (ARR 1.92, 95% CI 1.62–2.27) and 50% (ARR 1.50, 95% CI 1.32–1.74) increased risk of pain or discomfort, respectively, when compared to isolated head injury patients. Patients injured in low falls showed an increase in the risk of reporting pain or discomfort from 24 to 36 months, while noncompensable patients showed a sustained reduction of pain until 24-months postinjury before an increase in risk of pain or discomfort at the 36-month follow-up. Patients working prior to injury continued to show significant improvement on this item until 24-months postinjury.

#### Anxiety or depression

As for the other items of the EQ-5D-3L, women, compensable patients, and those with lower education or preinjury disability were at increased risk of reporting anxiety or depression problems at follow-up (Fig 3 and S5 Table). The risk of reporting anxiety or depression was 20% (ARR 1.20, 95% CI 1.10–1.31) higher for women compared to men and 58% (ARR 1.58, 95% CI 1.37–1.81) higher for compensable patients compared to noncompensable patients. The risk of reporting anxiety and depression was 28% (ARR 1.28, 95% CI 1.09–1.51) higher for patients who completed high school, 29% (ARR 1.29, 95% CI 1.12–1.48) higher for patients with a diploma or certificate and 32% (ARR 1.32, 95% CI 1.14–1.53) for patients who did not complete high school, when compared to patient with a university level of education (S5 Table). Age demonstrated a different pattern from the other EQ-5D-3L items, however, with patients aged 25 to 34 (ARR 1.21, 95% CI 1.05–1.39), 35–44 (ARR 1.19, 95% CI 1.03–1.39) and 44–54 years (ARR 1.20, 95% CI 1.04–1.39) experiencing a greater risk of anxiety or depression relative to the youngest age group. In contrast, patients 75 years and over experienced a 24% (ARR 0.76, 95% CI 0.64–0.91) lower risk of anxiety or depression than the youngest age group (Fig 3 and S5 Table). Working prior to injury was associated with a lower risk of reporting anxiety or depression by 20% (ARR 0.80, 95% CI 0.72–0.88). Patients in higher quintiles of socioeconomic advantage experienced a lower risk of reporting problems with anxiety or depression than those in the lowest quintile—by 13% (ARR 0.87, 95% CI 0.75–1.00) for quintile 2, 11% (ARR 0.89, 95% CI 0.78–1.00) for quintile 3, 14% (ARR 0.86, 95% CI 0.76–0.97) for quintile 4, and 17% (ARR 0.83, 95% CI 0.74–0.95) for quintile 5. Patients injured through interpersonal violence or self-harm demonstrated a 40% (ARR 1.40, 95% CI 1.20–1.63) increase in the risk of this outcome when compared to patients injured in unintentional events (Fig 3 and S5 Table). A preexisting mental health, alcohol, or drug condition was associated with a 13% (ARR 1.13, 95% CI 1.01–1.26) increase in the risk of reporting anxiety or depression at follow-up (S5 Table). There was no difference in change over time in the prevalence of anxiety or depression between subgroups of each covariate.

## Discussion

To our knowledge, we have conducted the largest, longitudinal study of health outcomes in seriously injured patients to date. Of the 2,757 hospitalised, adult major trauma patients, approximately 1 in 5 had died by 36-months postinjury, and the prevalence of persistent problems in survivors remained high for each of the EQ-5D-3L items. Forty percent of deaths in the cohort occurred after hospital discharge. Improvement to 24-months postinjury was evident for most items of the EQ-5D-3L, while improvement from 24- to 36-months postinjury was observed only for the usual activities item. Notably, the EQ-5D-3L summary score declined from 24- to 36-months postinjury, driven by an increase in reporting of pain or discomfort. Three factors were important predictors of outcome for all items of the EQ-5D-3L, age, compensable status, and level of education, while the nature of injuries sustained, gender, preinjury employment, and level of socioeconomic disadvantage were important predictors of problems on many of the 5 EQ-5D-3L items.

Claiming compensation from the state’s third-party, no-fault insurers for transport and work-related injury was a predictor of poorer outcome, even after adjusting for potential confounders such as age, socioeconomic status, and the nature of injuries sustained. The finding of poorer long-term outcomes in compensable patient groups is not new [8,32–34]. In our cohort, compensable patients had a higher prevalence of problems on each item of the EQ-5D-3L at each time point after injury than noncompensable patients. However, the compensable group did continue to improve beyond 24-months postinjury in self-care and usual activities, whereas noncompensable patients showed either no change or a decline in outcome from 24- to 36-months postinjury (S1 to S5 Tables). These findings suggest that the recovery process for compensable patients is slower than for other patients. The difference in prevalence of problems in usual activities between compensable and noncompensable cases halved over the study timeframe. The reasons for the differences in recovery rates between compensable and noncompensable patients is not fully known, but a number of factors may contribute. The complexity of navigating compensation agency processes has been suggested as a factor that may contribute to the disparities in health outcomes observed, with previous studies highlighting the association between stressful interactions with compensation agencies and poorer patient-reported outcomes [35,36]. Others have raised the potential for illness behaviour directed towards secondary gain from compensation agencies [37,38].

Increasing age has been identified previously as a predictor of poorer health status and functional outcome [8,10,11,14,33,39], although we found that the relationship between age and outcome differed between the EQ-5D-3L items. There was a dose-response-like increase in adjusted risk of reporting problems with each increase in age group for all items except the anxiety and depression item, for which older patients experienced significantly lower adjusted risk of reporting problems compared to the younger patients. The lower rates of anxiety and depression in older patients may reflect different life circumstances and pressures, and an overall lower risk of mental health issues in older age groups. Notably, recovery was poorer for older patients for most EQ-5D items (S1 to S5 Tables). As the trauma population ages, the burden of injury and the load on rehabilitation and disability services is expected to increase. Identification of interventions to improve outcomes in older trauma patients will be needed to mitigate this.

Our findings, combined with the results of previous studies [8,12,14,40], confirm that socioeconomic status is an important factor in determining outcomes following injury. Less than a university level of education was associated with greater adjusted risk of poorer outcomes on all EQ-5D-3L items, and there was a dose-response-like relationship between socioeconomic disadvantage and most outcomes. Studies of trauma patients have shown low health literacy, particularly in disadvantaged groups, with limited understanding of injuries and postsurgical instructions [41,42]. Others have shown poorer outcomes in surgical patients with lower health literacy [43]. Knowing what could improve one’s outcome and how to access appropriate services in a complex healthcare system is not easy for any patient and is especially challenging for those with low health literacy. These factors, along with service delays, waiting lists, and lack of financial resources in disadvantaged areas, could explain the disparity in outcomes by education level and socioeconomic status. Reducing health literacy demands on patients and improving health literacy amongst trauma populations could lead to better outcomes for patients [44].

Patients with a spinal cord injury demonstrated significantly poorer outcomes. The findings further highlight the life impacts of spinal cord injuries and support the need for continued investment in prevention and treatment options for this devastating injury type. In contrast, patients with isolated chest or abdominal injuries experienced better outcomes, although 30% continued to report problems on 3 of the 5 EQ-5D-3L items at 36-months postinjury.

In our study, the ARR of reporting problems on the mobility, usual activities, pain or discomfort, and anxiety or depression items was significantly higher for women compared to men. This difference has been identified in numerous trauma outcome studies [8,12,14,33,45,46] and remains unexplained. Differences in the psychological impact of the injury, and the influence of different social roles and responsibilities, have been suggested. Notably, intent of injury and the presence of a preexisting mental health, drug, or alcohol problem were not predictors of any outcome other than anxiety or depression. The prevalence of anxiety or depression problems was 15% to 20% higher in victims of interpersonal violence and 8% to 10% higher in patients injured through self-harm when compared to patients injured in unintentional events. These findings highlight the complex psychosocial needs of patients injured in intentional events.

The key strengths of this study were the large sample size, population-based design, prospective data collection, standardised approach to follow-up, use of a validated and recommended measure of health status in trauma, and high rates of follow-up. Additionally, the volume of missing data was low. Values were likely to be missing at random, allowing use of multiple imputation methods, and the complete case analyses were consistent with the results presented in this study from the imputed datasets. Nevertheless, there was responder bias, with patients injured in intentional events, younger, and less seriously injured patients being less likely to participate; therefore, these patient subgroups were underrepresented in the study findings.

In conclusion, this large-population cohort study of hospitalised major trauma patients demonstrates ongoing and dynamic differences in recovery trajectories across injury groups to 3-years postinjury. These findings highlight the high prevalence of ongoing problems following serious injury and have significant implications for trauma system design, injury rehabilitation programs, compensation schemes, and estimates of injury burden. Investment in interventions designed to prevent major trauma overall, and to reduce the impact of injury, is clearly needed.

## Supporting information

S1 Table Number of patients, prevalence, and predictors of reporting some/severe problems on the mobility item of the EQ-5D-3L—results of multivariable longitudinal analyses. (DOCX)

S2 Table Number of patients, prevalence, and predictors of reporting some/severe problems on the self-care item of the EQ-5D-3L—results of multivariable longitudinal analyses. (DOCX)

S3 Table Number of patients, prevalence, and predictors of reporting some/severe problems on the usual activities item of the EQ-5D-3L—results of multivariable longitudinal analyses. (DOCX)

S4 Table Number of patients, prevalence, and predictors of reporting some/severe problems on the pain or discomfort item of the EQ-5D-3L—results of multivariable longitudinal analyses. (DOCX)

S5 Table Number of patients, prevalence, and predictors of reporting some/severe problems on the anxiety or depression item of the EQ-5D-3L—results of multivariable longitudinal analyses. (DOCX)

S1 Text STROBE checklist. (DOCX)

## Abbreviations

ARIA: Accessibility/Remoteness Index of Australia
ARR: adjusted relative risk
CCI: Charlson Comorbidity Index
CI: confidence intervals
EQ-5D-3L: 3-level EuroQol 5 dimensions questionnaire
ICD-10-AM: 10th Revision of the International Classification of Diseases—Australian Modification
IRSAD: Index of Relative Socioeconomic Advantage and Disadvantage
RESTORE: REcovery after Serious Trauma—Outcomes, Resource use, and patient Experiences
RR: relative risk
SD: standard deviation
TAC: Transport Accident Commission
VSTR: Victorian State Trauma Registry
