# Supplementary material for: Long-term health status and trajectories of seriously injured patients: A population-based longitudinal study
Source: PLoS Med. 2017 Jul 5;14(7):e1002322. doi: 10.1371/journal.pmed.1002322 (PMC5497942; doi:10.1371/journal.pmed.1002322)
Supplement: S1 Table — (DOCX) [file pmed.1002322.s001.docx]

**S1 Table: Number of patients, prevalence and predictors of reporting some/severe problems on the mobility item of the EQ-5D-3L - results of multivariable longitudinal analyses**

|  | **6 months**  **N = 1962** | | **12 months**  **N = 1959** | | **24 months**  **N = 1891** | | **36 months**  **N = 1632** | | **Adjusted relative risk* (95% CI)** | **p-value** |
| --- | --- | --- | --- | --- | --- | --- | --- | --- | --- | --- |
|  | **n** | % problems in each group  (95% CI) | **n** | % problems in each group  (95% CI) | **n** | % problems in each group  (95% CI) | **n** | % problems in each group  (95% CI) |  |  |
| **Sex** |  |  |  |  |  |  |  |  |  |  |
| Male | 548 | 38.7 (36.1, 41.2) | 512 | 36.1 (33.6, 38.6) | 441 | 32.2 (29.7, 34.7) | 383 | 32.1 (29.4, 34.8) | Reference | 0.05 |
| Female | 300 | 55.2 (50.9, 59.4) | 271 | 50.3 (46.0, 54.6) | 263 | 50.6 (46.2, 55.0) | 221 | 50.5 (45.7, 55.2) | 1.10 (1.00, 1.21) |  |
| **Age group** |  |  |  |  |  |  |  |  |  |  |
| 18-24 years | 80 | 29.5 (24.2, 35.3) | 67 | 25.6 (20.4, 31.3) | 54 | 20.9 (16.1, 26.4) | 48 | 22.3 (16.9, 28.5) | Reference | <0.001 |
| 25-34 years | 97 | 35.3 (29.6, 41.2) | 90 | 31.9 (26.5, 37.7) | 77 | 28.6 (23.3, 34.4) | 59 | 25.2 (19.8, 31.3) | 1.25 (1.01, 1.54) |  |
| 35-44 years | 95 | 33.6 (28.1, 39.4) | 98 | 34.3 (28.8, 40.1) | 76 | 27.6 (22.4, 33.3) | 65 | 27.3 (21.8, 33.4) | 1.28 (1.03, 1.58) |  |
| 45-54 years | 124 | 42.0 (36.3, 47.9) | 104 | 35.6 (30.1, 41.4) | 95 | 33.5 (28.0, 39.3) | 84 | 32.2 (26.6, 38.2) | 1.48 (1.21, 1.82) |  |
| 55-64 years | 112 | 39.9 (34.1, 45.8) | 102 | 35.8 (30.2, 41.7) | 94 | 33.8 (28.3, 39.7) | 95 | 37.4 (31.4, 43.7) | 1.43 (1.16, 1.76) |  |
| 65-74 years | 104 | 46.2 (39.6, 53.0) | 101 | 44.9 (38.3, 51.6) | 82 | 37.3 (30.9, 44.0) | 91 | 47.9 (40.6, 55.2) | 1.54 (1.25, 1.91) |  |
| 75+ years | 236 | 71.1 (65.9, 75.9) | 221 | 67.6 (62.2, 72.6) | 226 | 73.6 (68.3, 78.5) | 162 | 67.5 (61.2, 73.4) | 2.05 (1.66, 2.53) |  |
| **Charlson comorbidity index** |  |  |  |  |  |  |  |  |  |  |
| 0 | 531 | 41.1 (38.4, 43.9) | 492 | 37.9 (35.2, 40.6) | 458 | 36.1 (33.4, 38.8) | 389 | 35.4 (32.5, 38.3) | Reference | 0.02 |
| 1 | 224 | 44.2 (39.8, 48.6) | 202 | 39.9 (35.6, 44.3) | 164 | 34.8 (30.5, 39.2) | 148 | 36.8 (32.1, 41.7) | 1.01 (0.89, 1.14) |  |
| 2+ | 93 | 56.7 (48.8, 64.4) | 89 | 57.8 (49.6, 65.7) | 82 | 54.7 (46.3, 62.8) | 67 | 51.5 (42.6, 60.4) | 1.21 (1.06, 1.39) |  |
| **Region** |  |  |  |  |  |  |  |  |  |  |
| Major cities | 607 | 44.5 (41.8, 47.2) | 549 | 40.7 (38.0, 43.3) | 503 | 38.7 (36.0, 41.4) | 418 | 37.4 (34.5, 40.3) | Reference | 0.84 |
| Regional or remote | 217 | 39.9 (35.7, 44.1) | 214 | 38.4 (25.3, 53.0) | 187 | 34.7 (30.7, 38.9) | 175 | 37.0 (32.6, 41.5) | 0.98 (0.82, 1.17) |  |
| **Major trauma service** |  |  |  |  |  |  |  |  |  |  |
| No | 123 | 43.3 (37.5, 49.3) | 127 | 40.5 (35.0, 46.1) | 126 | 40.4 (34.9, 46.1) | 85 | 37.6 (31.3, 44.3) | Reference | 0.04 |
| Yes | 725 | 43.2 (40.8, 45.6) | 656 | 39.9 (37.5, 42.3) | 578 | 36.6 (34.2, 39.0) | 519 | 36.9 (34.4, 39.5) | 1.13 (1.01, 1.27) |  |
| **Cause of injury** |  |  |  |  |  |  |  |  |  |  |
| Motor vehicle occupant | 246 | 50.3 (45.8, 54.8) | 230 | 47.1 (42.6, 51.7) | 201 | 43.9 (39.3, 48.6) | 180 | 46.3 (41.2, 51.4) | Reference | <0.001 |
| Motorcyclist | 78 | 37.3 (30.7, 44.3) | 79 | 37.4 (30.9, 44.3) | 70 | 33.3 (27.0, 40.1) | 62 | 34.1 (27.2, 41.4) | 0.94 (0.79, 1.11) |  |
| Pedal cyclist/pedestrian | 86 | 38.1 (31.7, 44.7) | 78 | 35.8 (29.4, 42.5) | 72 | 32.4 (26.3, 39.0) | 62 | 31.3 (24.9, 38.3) | 0.88 (0.75, 1.03) |  |
| Low fall (≤ 1m) | 244 | 61.3 (56.3, 66.1) | 221 | 56.5 (51.4, 61.5) | 208 | 57.6 (52.3, 62.8) | 162 | 57.0 (51.1, 62.9) | 1.38 (1.13, 1.70) |  |
| High fall (>1m) | 72 | 27.9 (22.5, 33.8) | 68 | 26.0 (20.8, 31.7) | 64 | 24.2 (19.1, 29.8) | 62 | 26.2 (20.7, 32.2) | 0.87 (0.71, 1.08) |  |
| Struck by/collision with person/object | 55 | 32.7 (25.7, 40.4) | 39 | 22.9 (16.9, 30.0) | 36 | 21.4 (15.5, 28.4) | 29 | 18.8 (13.0, 25.9) | 0.80 (0.60, 1.05) |  |
| Other | 67 | 31.3 (25.2, 38.0) | 68 | 31.1 (25.0, 37.6) | 53 | 25.6 (19.8, 32.1) | 47 | 25.0 (19.0, 31.8) | 0.97 (0.78, 1.21) |  |
| **Intent** |  |  |  |  |  |  |  |  |  |  |
| Unintentional | 781 | 44.1 (41.8, 46.5) | 722 | 40.9 (38.6, 43.2) | 657 | 38.5 36.2, 40.8) | 563 | 38.2 (35.7, 40.7) | Reference | 0.05 |
| Intentional | 58 | 33.5 (26.5, 41.1) | 52 | 29.7 (23.1, 37.1) | 41 | 24.9 (18.5, 32.2) | 33 | 23.4 (16.7, 31.3) | 1.27 (1.00, 1.61) |  |
| **Compensable status** |  |  |  |  |  |  |  |  |  |  |
| Non-compensable | 418 | 38.6 (35.7, 41.6) | 378 | 34.9 (32.1, 37.8) | 345 | 33.1 (30.3, 36.1) | 288 | 32.1 (29.1, 35.3) | Reference | <0.001 |
| Compensable | 422 | 48.7 (45.4, 52.1) | 395 | 45.8 (42.5, 49.2) | 351 | 42.0 (38.6, 45.4) | 311 | 42.8 (39.2, 46.5) | 1.92 (1.64, 2.24) |  |
| **Working Prior to injury** |  |  |  |  |  |  |  |  |  |  |
| No | 461 | 59.2 (55.6, 62.7) | 426 | 54.8 (51.2, 58.3) | 397 | 53.8 (50.1, 57.4) | 325 | 54.3 (50.2, 58.3) | Reference | <0.001 |
| Yes | 387 | 32.8 (30.1, 35.5) | 356 | 30.2 (27.6, 32.9) | 305 | 26.5 (24.1, 29.2) | 277 | 26.9 (24.3, 29.8) | 0.77 (0.68, 0.87) |  |
| **Pre-injury disability level** |  |  |  |  |  |  |  |  |  |  |
| None | 546 | 35.8 (33.4, 38.3) | 503 | 33.1 (30.7, 35.5) | 460 | 31.1 (28.7, 33.5) | 403 | 31.0 (28.5, 33.6) | Reference | <0.001 |
| Mild | 156 | 65.3 (58.9, 71.3) | 157 | 66.0 (59.6, 72.0) | 133 | 59.4 (52.6, 65.9) | 112 | 60.9 (53.4, 68.0) | 1.50 (1.34, 1.67) |  |
| Moderate | 94 | 77.7 (69.2, 84.8) | 81 | 66.9 (57.8, 75.2) | 72 | 62.1 (52.6, 70.9) | 55 | 61.1 (50.3, 71.2) | 1.56 (1.37, 1.78) |  |
| Marked/severe | 48 | 70.6 (58.3, 81.0) | 35 | 50.7 (38.4, 63.0) | 33 | 54.1 (40.8, 66.9) | 30 | 57.7 (43.2, 71.3) | 1.40 (1.16, 1.69) |  |
| **Socioeconomic status (IRSAD)** |  |  |  |  |  |  |  |  |  |  |
| 1 – most disadvantaged | 118 | 50.0 (43.4, 56.6) | 116 | 48.1 (41.7, 54.6) | 107 | 46.1 (39.6, 52.8) | 88 | 45.4 (38.2, 52.6) | Reference | 0.07 |
| 2 | 119 | 46.9 (40.6, 53.2) | 118 | 47.8 (41.4, 54.2) | 95 | 41.3 (34.9, 48.0) | 78 | 38.6 (31.9, 45.7) | 0.94 (0.81, 1.10) |  |
| 3 | 132 | 37.7 (32.6, 43.0) | 119 | 33.9 (29.0, 39.1) | 114 | 32.0 (27.2, 37.1) | 106 | 35.5 (30.0, 41.2) | 0.85 (0.73, 0.98) |  |
| 4 | 239 | 43.4 (39.2, 47.6) | 213 | 38.3 (34.3, 42.5) | 202 | 38.0 (33.8, 42.2) | 181 | 38.8 (34.3, 43.3) | 0.89 (0.78, 1.02) |  |
| 5 – most advantaged | 216 | 41.8 (37.5, 46.2) | 197 | 38.5 (34.2, 42.8) | 172 | 35.2 (30.9, 39.6) | 140 | 32.6 (28.2, 37.3) | 0.83 (0.72, 0.95) |  |
| **Nature of injury** |  |  |  |  |  |  |  |  |  |  |
| Isolated head injury | 143 | 52.4 (46.3, 58.4) | 128 | 47.1 (41.0, 53.2) | 109 | 43.3 (37.1, 49.6) | 95 | 44.4 (37.6, 51.3) | Reference | <0.001 |
| Head and other injuries | 191 | 41.3 (36.8, 46.0) | 176 | 39.7 (35.1, 44.5) | 154 | 35.6 (31.1, 40.3) | 128 | 33.7 (28.9, 38.7) | 0.99 (0.87, 1.14) |  |
| Spinal cord injury | 45 | 77.6 (64.7, 87.5) | 47 | 79.7 (67.2, 89.0) | 46 | 79.3 (66.6, 88.8) | 41 | 75.9 (62.4, 86.5) | 2.63 (2.06, 3.35) |  |
| Orthopaedic injuries only | 85 | 44.5 (37.3, 51.9) | 83 | 41.9 (35.0, 49.1) | 74 | 39.0 (32.0, 46.3) | 63 | 38.4 (30.9, 46.3) | 1.05 (0.89, 1.23) |  |
| Chest/abdominal injuries alone | 39 | 21.8 (16.0, 28.6) | 41 | 22.2 (16.4, 28.8) | 40 | 23.7 (17.5, 30.8) | 33 | 23.4 (16.7, 31.3) | 0.65 (0.52, 0.81) |  |
| Chest/abdominal and other injuries | 220 | 43.6 (39.2, 48.0) | 195 | 38.4 (34.1, 42.8) | 178 | 35.2 (31.0, 39.5) | 153 | 35.4 (30.9, 40.1) | 0.97 (0.84, 1.13) |  |
| Other multi-trauma and burns | 125 | 42.5 (36.8, 48.4) | 113 | 38.4 (32.8, 44.3) | 103 | 36.4 (30.8, 42.3) | 91 | 36.8 (30.8, 43.2) | 1.05 (0.90, 1.22) |  |
| **Education** |  |  |  |  |  |  |  |  |  |  |
| University degree | 90 | 28.2 (23.3, 33.5) | 81 | 25.9 (21.1, 31.1) | 77 | 25.3 (20.5, 30.5) | 66 | 23.7 (18.8, 29.1) | Reference | 0.01 |
| Completed high school | 74 | 33.3 (27.2, 40.0) | 67 | 31.2 (25.0, 37.8) | 67 | 32.8 (26.4, 39.7) | 54 | 30.5 (23.8, 37.9) | 1.16 (0.94, 1.43) |  |
| Diploma or certificate | 232 | 40.4 (36.4, 44.6) | 212 | 37.7 (33.6, 41.8) | 188 | 33.5 (29.6, 37.6) | 177 | 35.6 (31.4, 40.0) | 1.28 (1.09, 1.51) |  |
| Did not complete high school | 350 | 51.9 (48.0, 55.7) | 327 | 46.9 (43.1, 50.6) | 291 | 43.8 (39.9, 47.6) | 235 | 42.5 (38.3, 46.7) | 1.29 (1.10, 1.52) |  |
| **Alcohol/mental health issues** |  |  |  |  |  |  |  |  |  |  |
| No | 613 | 43.2 (40.6, 45.8) | 564 | 39.9 (37.4, 42.6) | 522 | 38.0 (35.4, 40.6) | 453 | 37.4 (34.7, 40.2) | Reference | 0.39 |
| Yes | 215 | 43.2 (38.8, 47.7) | 196 | 39.6 (35.3, 44.1) | 152 | 32.9 (28.6, 37.4) | 132 | 34.1 (29.4, 39.1) | 0.94 (0.83, 1.08) |  |

*Model adjusted for each item presented in this table
