# Supplementary material for: Long-term health status and trajectories of seriously injured patients: A population-based longitudinal study
Source: PLoS Med. 2017 Jul 5;14(7):e1002322. doi: 10.1371/journal.pmed.1002322 (PMC5497942; doi:10.1371/journal.pmed.1002322)
Supplement: S2 Table — (DOCX) [file pmed.1002322.s002.docx]

**S2 Table: Number of patients, prevalence and predictors of reporting some/severe problems on the self-care item of the EQ-5D-3L - results of multivariable longitudinal analyses**

|  | **6 months**  **N = 1962** | | **12 months**  **N = 1959** | | **24 months**  **N = 1889** | | **36 months**  **N = 1631** | | **Adjusted relative risk* (95% CI)** | **p-value** |
| --- | --- | --- | --- | --- | --- | --- | --- | --- | --- | --- |
|  | **n** | % problems in each group  (95% CI) | **n** | % problems in each group  (95% CI) | **n** | % problems in each group  (95% CI) | **n** | % problems in each group  (95% CI) |  |  |
| **Sex** |  |  |  |  |  |  |  |  |  |  |
| Male | 335 | 23.6 (21.4, 25.9) | 286 | 20.1 (18.1, 22.3) | 273 | 19.9 (17.9, 22.2) | 204 | 17.1 (15.0, 19.4) | Reference | 0.07 |
| Female | 199 | 36.7 (32.6, 40.9) | 174 | 32.3 (28.3, 36.4) | 167 | 32.1 (28.1, 36.3) | 138 | 31.5 (27.2, 36.1) | 1.13 (0.99, 1.29) |  |
| **Age** |  |  |  |  |  |  |  |  |  |  |
| 18-24 years | 36 | 13.3 (9.5, 17.9) | 33 | 12.6 (8.8, 17.2) | 29 | 11.2 (7.7, 15.7) | 16 | 7.4 (4.3, 11.8) | Reference | <0.001 |
| 25-34 years | 54 | 19.6 (15.1, 24.8) | 44 | 15.6 (11.6, 20.4) | 43 | 16.0 (11.8, 20.9) | 26 | 11.1 (7.4, 15.9) | 1.43 (1.05, 1.96) |  |
| 35-44 years | 57 | 20.1 (15.6, 25.2) | 46 | 16.1 (12.0, 20.9) | 44 | 16.0 (11.9, 20.9) | 31 | 13.0 (9.0, 18.0) | 1.50 (1.09, 2.06) |  |
| 45-54 years | 77 | 26.1 (21.2, 31.5) | 65 | 22.3 (17.6, 27.5) | 56 | 19.7 (15.3, 24.8) | 49 | 18.8 (14.3, 24.1) | 1.93 (1.42, 2.63) |  |
| 55-64 years | 77 | 27.5 (22.4, 33.1) | 58 | 20.4 (15.8, 25.5) | 57 | 20.5 (15.9, 25.7) | 50 | 19.7 (15.0, 25.1) | 1.79 (1.31, 2.45) |  |
| 65-74 years | 64 | 28.4 (22.6, 34.8) | 60 | 26.7 (21.0, 33.0) | 58 | 26.6 (20.9, 33.0) | 48 | 25.3 (19.3, 32.1) | 1.94 (1.39, 2.70) |  |
| 75+ years | 169 | 50.9 (45.4, 56.4) | 154 | 47.1 (41.6, 52.7) | 153 | 49.8 (44.1, 55.6) | 122 | 50.8 (44.3, 57.3) | 2.75 (1.99, 3.80) |  |
| **Charlson comorbidity index** |  |  |  |  |  |  |  |  |  |  |
| 0 | 334 | 25.9 (23.5, 28.4) | 282 | 21.7 (19.5, 24.1) | 268 | 21.1 (18.9, 23.5) | 204 | 18.6 (16.3, 21.0) | Reference |  |
| 1 | 137 | 27.0 (23.2, 31.1) | 117 | 23.1 (19.5, 27.0) | 113 | 24.0 (20.2, 28.2) | 85 | 21.2 (17.3, 25.5) | 1.07 (0.90, 1.27) | 0.001 |
| 2+ | 63 | 38.4 (30.9, 46.3) | 61 | 39.6 (31.8, 47.8) | 59 | 39.3 (31.5, 47.6) | 53 | 40.8 (32.2, 49.7) | 1.46 (1.20, 1.77) |  |
| **Region** |  |  |  |  |  |  |  |  |  |  |
| Major cities | 385 | 28.2 (25.8, 30.7) | 341 | 25.3 (23.0, 27.7) | 315 | 24.321.9, 26.7) | 244 | 21.8 (19.4, 24.4) | Reference | 0.96 |
| Regional or remote | 136 | 25.0 (21.4, 28.9) | 108 | 19.4 (16.2, 22.9) | 114 | 21.2 (17.8, 24.8) | 86 | 18.2 (14.8, 22.0) | 0.99 (0.76, 1.29) |  |
| **Major trauma service** |  |  |  |  |  |  |  |  |  |  |
| No | 90 | 31.7 (26.3, 37.4) | 85 | 27.1 (22.2, 32.3) | 89 | 28.6 (23.7, 34.0) | 59 | 26.1 (20.5, 32.3) | Reference | 0.15 |
| Yes | 444 | 26.5 (24.4, 28.6) | 375 | 22.8 (20.8, 24.9) | 351 | 22.2 (20.2, 24.4) | 283 | 20.1 (18.1, 22.3) | 1.12 (0.96, 1.31) |  |
| **Cause of injury** |  |  |  |  |  |  |  |  |  |  |
| Motor vehicle occupant | 140 | 28.7 (24.7, 32.9) | 129 | 26.4 (22.6, 30.6) | 118 | 25.8 (21.8, 30.0) | 91 | 23.5 (19.3, 28.0) | Reference | <0.001 |
| Motorcyclist | 56 | 26.7 (20.8, 33.2) | 38 | 18.0 (13.1, 23.9) | 39 | 18.6 (13.6, 24.5) | 31 | 17.0 (11.9, 23.3) | 0.95 (0.74, 1.21) |  |
| Pedal cyclist/pedestrian | 48 | 21.2 (16.1, 27.2) | 39 | 17.9 (13.0, 23.6) | 41 | 18.6 (13.7, 24.3) | 34 | 17.2 (12.2, 23.2) | 0.86 (0.67, 1.10) |  |
| Low fall (≤ 1m) | 180 | 45.2 (40.3, 50.3) | 163 | 41.7 (36.8, 46.8) | 163 | 45.2 (40.0, 50.4) | 120 | 42.3 (36.4, 48.2) | 1.57 (1.17, 2.11) |  |
| High fall (>1m) | 37 | 14.3 (10.3, 19.2) | 33 | 12.6 (8.8, 17.2) | 25 | 9.5 (6.2, 13.7) | 23 | 9.7 (6.3, 14.2) | 0.67 (0.49, 0.91) |  |
| Struck by/collision with person/object | 26 | 15.5 (10.4, 21.8) | 22 | 12.9 (8.3, 18.9) | 20 | 11.9 (7.4, 17.8) | 17 | 11.0 (6.6, 17.1) | 0.75 (0.51, 1.09) |  |
| Other | 47 | 22.0 (16.6, 28.1) | 36 | 16.4 (11.8, 22.0) | 34 | 16.4 (11.7, 22.2) | 26 | 13.8 (9.2, 19.6) | 1.06 (0.78, 1.43) |  |
| **Intent** |  |  |  |  |  |  |  |  |  |  |
| Unintentional | 498 | 28.1 (26.1, 30.3) | 432 | 24.5 (22.5, 26.6) | 411 | 24.1 (22.1, 26.2) | 321 | 21.8 (19.7, 24.0) | Reference | 0.44 |
| Intentional | 32 | 18.5 (13.0, 25.1) | 23 | 13.1 (8.5, 19.1) | 26 | 15.8 (10.6, 22.2) | 16 | 11.4 (6.6, 17.8) | 1.14 (0.81, 1.60) |  |
| **Compensable status** |  |  |  |  |  |  |  |  |  |  |
| Non-compensable | 278 | 25.7 (23.1, 28.4) | 242 | 22.4 (19.9, 24.9) | 231 | 22.2 (19.7, 24.8) | 185 | 20.6 (18.0, 23.4) | Reference | <0.001 |
| Compensable | 249 | 28.8 (25.8, 31.9) | 213 | 24.7 (21.9, 27.7) | 205 | 24.6 (21.7, 27.6) | 155 | 21.4 (18.4, 24.5) | 1.97 (1.57, 2.47) |  |
| **Working prior to injury** |  |  |  |  |  |  |  |  |  |  |
| No | 298 | 38.3 (34.9, 41.8) | 273 | 35.1 (31.7, 38.6) | 280 | 38.0 (34.4, 41.6) | 208 | 34.8 (31.0, 38.8) | Reference | 0.01 |
| Yes | 236 | 20.0 (17.7, 22.4) | 186 | 15.8 (13.7, 18.0) | 157 | 13.7 (11.8, 15.9) | 132 | 12.8 (10.8, 15.0) | 0.78 (0.65, 0.93) |  |
| **Pre-injury disability level** |  |  |  |  |  |  |  |  |  |  |
| None | 309 | 20.3 (18.3, 22.4) | 269 | 17.7 (15.8, 19.7) | 261 | 17.6 (15.7, 19.7) | 209 | 16.1 (14.1, 18.2) | Reference | <0.001 |
| Mild | 110 | 46.0 (39.6, 52.6) | 94 | 39.5 (33.2, 46.0) | 83 | 37.4 (31.0, 44.1) | 68 | 37.2 (30.1, 44.6) | 1.60 (1.36, 1.89) |  |
| Moderate | 72 | 59.0 (49.7, 67.8) | 59 | 48.8 (39.6, 58.0) | 63 | 54.3 (44.8, 63.6) | 42 | 46.7 (36.1, 57.5) | 2.09 (1.71, 2.54) |  |
| Marked/severe | 39 | 57.4 (44.8, 69.3) | 32 | 46.4 (34.3, 58.8) | 27 | 44.3 (31.5, 57.6) | 20 | 38.5 (25.3, 53.0) | 1.99 (1.58, 2.52) |  |
| **Socioeconomic status (IRSAD)** |  |  |  |  |  |  |  |  |  |  |
| 1 – most disadvantaged | 80 | 33.9 (27.9, 40.3) | 67 | 27.8 (22.2, 33.9) | 68 | 29.3 (23.5, 35.6) | 59 | 30.4 (24.0, 37.4) | Reference | 0.05 |
| 2 | 72 | 28.4 (22.9, 34.3) | 61 | 24.7 (19.4, 30.6) | 56 | 24.4 (18.9, 30.4) | 45 | 22.3 (16.7, 28.6) | 0.80 (0.64, 1.01) |  |
| 3 | 84 | 24.1 (19.7, 28.9) | 69 | 19.7 (15.6, 24.2) | 62 | 17.5 (13.7, 21.8) | 45 | 15.1 (11.2, 19.6) | 0.76 (0.61, 0.94) |  |
| 4 | 159 | 28.9 (25.1, 32.8) | 139 | 25.0 (21.5, 28.8) | 127 | 23.9 (20.3, 27.7) | 101 | 21.7 (18.0, 25.7) | 0.88 (0.73, 1.07) |  |
| 5 – most advantaged | 126 | 24.3 (20.7, 28.3) | 113 | 22.1 (18.6, 25.9) | 116 | 23.7 (20.0, 27.7) | 80 | 18.7 (15.1, 22.7) | 0.78 (0.64, 0.95) |  |
| **Nature of injury** |  |  |  |  |  |  |  |  |  |  |
| Isolated head injury | 98 | 35.9 (30.2, 41.9) | 91 | 33.5 (27.9, 39.4) | 84 | 33.5 (27.7, 39.7) | 73 | 34.1 (27.8, 40.9) | Reference | <0.001 |
| Head and other injuries | 114 | 24.7 (20.9, 28.9) | 97 | 21.9 (18.1, 26.0) | 93 | 21.5 (17.7, 25.7) | 69 | 18.2 (14.4, 22.4) | 0.93 (0.76, 1.13) |  |
| Spinal cord injury | 39 | 67.2 (53.7, 79.0) | 31 | 52.5 (39.1, 65.7) | 27 | 46.6 (33.3, 60.1) | 30 | 55.6 (41.4, 69.1) | 3.57 (2.60, 4.92) |  |
| Orthopaedic injuries only | 58 | 30.4 (23.9, 37.4) | 41 | 20.7 (15.3, 27.0) | 48 | 25.3 (19.3, 32.1) | 32 | 19.5 (13.7, 26.4) | 0.99 (0.79, 1.23) |  |
| Chest/abdominal injuries alone | 22 | 12.3 (7.9, 18.0) | 29 | 15.7 (10.8, 21.7) | 27 | 16.0 (10.8, 22.4) | 18 | 12.8 (7.7, 19.4) | 0.66 (0.49, 0.88) |  |
| Chest/abdominal and other injuries | 131 | 25.9 (22.1, 29.9) | 104 | 20.5 (17.0, 24.2) | 105 | 20.8 (17.3, 24.5) | 73 | 16.9 (13.5, 20.8) | 0.93 (0.75, 1.15) |  |
| Other multi-trauma and burns | 72 | 24.5 (19.7, 29.8) | 67 | 22.8 (18.1, 28.0) | 56 | 19.8 (15.3, 24.9) | 47 | 19.0 (14.3, 24.4) | 0.97 (0.77, 1.21) |  |
| **Education** |  |  |  |  |  |  |  |  |  |  |
| University degree | 58 | 18.2 (14.1, 22.9) | 39 | 12.5 (9.0, 16.6) | 34 | 11.2 (7.8, 15.2) | 29 | 10.4 (7.1, 14.6) | Reference | 0.01 |
| Completed high school | 36 | 16.2 (11.6, 21.7) | 38 | 17.7 (12.8, 23.4) | 31 | 15.2 (10.6, 20.9) | 26 | 14.7 (9.8, 20.8) | 1.21 (0.90, 1.62) |  |
| Diploma or certificate | 152 | 26.5 (22.9, 30.3) | 113 | 20.1 (16.8, 23.6) | 111 | 19.8 (16.6, 23.3) | 83 | 16.7 (13.6, 20.3) | 1.43 (1.13, 1.82) |  |
| Did not complete high school | 207 | 30.7 (27.2, 34.3) | 189 | 27.1 (23.8, 30.5) | 191 | 28.8 (25.3, 32.4) | 147 | 26.6 (22.9, 30.5) | 1.45 (1.14, 1.84) |  |
| **Alcohol/mental health issues** |  |  |  |  |  |  |  |  |  |  |
| No | 393 | 27.7 (25.4, 30.1) | 334 | 23.7 (21.5, 26.0) | 316 | 23.0 (20.8, 25.3) | 252 | 20.8 (18.6, 23.2) | Reference | 0.47 |
| Yes | 127 | 25.5 (21.7, 29.6) | 110 | 22.2 (18.6, 26.1) | 104 | 22.6 (18.9, 26.7) | 80 | 20.7 (16.7, 25.1) | 0.94 (0.78, 1.12) |  |

*Model adjusted for each item presented in this table
