# Supplementary material for: Long-term health status and trajectories of seriously injured patients: A population-based longitudinal study
Source: PLoS Med. 2017 Jul 5;14(7):e1002322. doi: 10.1371/journal.pmed.1002322 (PMC5497942; doi:10.1371/journal.pmed.1002322)
Supplement: S3 Table — (DOCX) [file pmed.1002322.s003.docx]

**S3 Table: Number of patients, prevalence and predictors of reporting some/severe problems on the usual activities item of the EQ-5D-3L - results of multivariable longitudinal analyses**

|  | **6 months**  **N = 1961** | | **12 months**  **N = 1957** | | **24 months**  **N = 1890** | | **36 months**  **N = 1632** | | **Adjusted relative risk* (95% CI)** | **p-value** |
| --- | --- | --- | --- | --- | --- | --- | --- | --- | --- | --- |
|  | **n** | % problems in each group  (95% CI) | **n** | % problems in each group  (95% CI) | **n** | % problems in each group  (95% CI) | **n** | % problems in each group  (95% CI) |  |  |
| **Sex** |  |  |  |  |  |  |  |  |  |  |
| Male | 831 | 58.7 (56.0, 61.2) | 714 | 50.4 (47.7, 53.0) | 630 | 46.0 (43.3, 48.7) | 511 | 42.8 (40.0, 45.7) | Reference | 0.003 |
| Female | 376 | 69.1 (65.0, 73.0) | 331 | 61.4 (57.2, 65.5) | 317 | 61.0 (56.6, 65.2) | 258 | 58.9 (54.1, 63.6) | 1.11 (1.04, 1.18) |  |
| **Age** |  |  |  |  |  |  |  |  |  |  |
| 18-24 years | 126 | 46.5 (40.4, 52.6) | 110 | 42.0 (35.9, 48.2) | 95 | 36.8 (30.9, 43.0) | 71 | 33.0 (26.8, 39.7) | Reference | <0.001 |
| 25-34 years | 159 | 57.8 (51.7, 63.7) | 137 | 48.8 (42.8, 54.8) | 122 | 45.4 (39.3, 51.5) | 81 | 34.6 (28.5, 41.1) | 1.20 (1.04, 1.38) |  |
| 35-44 years | 163 | 57.4 (51.4, 63.2) | 149 | 52.1 (46.1, 58.0) | 134 | 48.9 (42.8, 55.0) | 101 | 42.4 (36.1, 49.0) | 1.30 (1.13, 1.49) |  |
| 45-54 years | 180 | 61.0 (55.2, 66.6) | 166 | 56.8 (51.0, 62.6) | 135 | 47.5 (41.6, 53.5) | 121 | 46.4 (40.2, 52.6) | 1.37 (1.19, 1.57) |  |
| 55-64 years | 192 | 68.8 (63.0, 74.2) | 159 | 55.8 (49.8, 61.6) | 139 | 50.0 (44.0, 56.0) | 133 | 52.4 (46.0, 58.6) | 1.47 (1.28, 1.68) |  |
| 65-74 years | 140 | 62.2 (55.5, 68.6) | 112 | 50.0 (43.3, 56.7) | 111 | 50.5 (43.7, 57.2) | 99 | 52.1 (44.8, 59.4) | 1.40 (1.20, 1.63) |  |
| 75+ years | 247 | 74.4 (69.3, 79.0) | 212 | 64.8 (59.4, 70.0) | 211 | 68.7 (63.2, 73.9) | 163 | 67.9 (61.6, 73.8) | 1.59 (1.37, 1.85) |  |
| **Charlson comorbidity index** |  |  |  |  |  |  |  |  |  |  |
| 0 | 774 | 59.9 (57.2, 62.6) | 657 | 50.7 (47.9, 53.4) | 598 | 47.1 (44.3, 49.9) | 482 | 43.8 (40.9, 46.8) | Reference | 0.03 |
| 1 | 322 | 63.6 (59.3, 67.8) | 288 | 56.9 (52.5, 61.3) | 253 | 53.7 (49.1, 58.3) | 203 | 50.5 (45.5, 55.5) | 1.06 (0.97, 1.15) |  |
| 2+ | 111 | 68.1 (60.4, 75.2) | 100 | 64.9 (56.8, 72.4) | 96 | 64.0 (55.8, 71.7) | 84 | 64.6 (55.8, 72.8) | 1.14 (1.03, 1.25) |  |
| **Region** |  |  |  |  |  |  |  |  |  |  |
| Major cities | 847 | 62.1 (59.5, 64.7) | 728 | 54.0 (51.3, 56.7) | 666 | 51.2 (48.5, 54.0) | 531 | 47.5 (44.5, 50.5) | Reference | 0.70 |
| Regional or remote | 329 | 60.6 (56.3, 64.7) | 293 | 52.6 (48.4, 56.8) | 259 | 48.1 (43.8, 52.5) | 221 | 46.7 (42.2, 51.3) | 0.98 (0.86, 1.10) |  |
| **Major trauma service** |  |  |  |  |  |  |  |  |  |  |
| No | 163 | 57.4 (51.4, 63.2) | 155 | 49.5 (43.8, 55.2) | 153 | 49.0 (43.4, 54.7) | 103 | 45.6 (39.0, 52.3) | Reference | 0.13 |
| Yes | 1044 | 62.3 (59.9, 64.6) | 890 | 54.1 (51.7, 56.6) | 794 | 50.3 (47.8, 52.8) | 666 | 47.4 (44.7, 50.0) | 1.07 (0.98, 1.17) |  |
| **Cause of injury** |  |  |  |  |  |  |  |  |  |  |
| Motor vehicle occupant | 346 | 70.8 (66.5, 74.8) | 304 | 62.4 (58.0, 66.7) | 280 | 61.1 (56.5, 65.6) | 220 | 56.6 (51.5, 61.5) | Reference | 0.001 |
| Motorcyclist | 132 | 62.9 (55.9, 69.4) | 113 | 53.6 (46.6, 60.4) | 104 | 49.8 (42.8, 56.7) | 79 | 43.4 (36.1, 50.9) | 0.94 (0.84, 1.05) |  |
| Pedal cyclist/pedestrian | 127 | 56.4 (49.7, 63.0) | 107 | 49.1 (42.3, 55.9) | 92 | 41.4 (34.9, 48.2) | 77 | 38.9 (32.1, 46.1) | 0.85 (0.76, 0.95) |  |
| Low fall (≤ 1m) | 270 | 68.0 (63.2, 72.6) | 230 | 58.8 (53.8, 63.7) | 227 | 62.9 (57.7, 67.9) | 175 | 61.6 (55.7, 67.3) | 1.13 (0.98, 1.32) |  |
| High fall (>1m) | 133 | 51.6 (45.3, 57.8) | 107 | 41.0 (35.0, 47.2) | 93 | 35.1 (29.4, 41.2) | 85 | 35.9 (29.8, 42.3) | 0.89 (0.77, 1.03) |  |
| Struck by/collision with person/object | 90 | 53.6 (45.7, 61.3) | 78 | 45.9 (38.2, 53.7) | 61 | 36.3 (29.0, 44.1) | 59 | 38.3 (30.6, 46.5) | 0.97 (0.82, 1.15) |  |
| Other | 109 | 50.9 (44.0, 57.8) | 106 | 48.4 (41.6, 55.2) | 90 | 43.5 (36.6, 50.5) | 74 | 39.4 (32.3, 46.7) | 1.02 (0.88, 1.18) |  |
| **Intent** |  |  |  |  |  |  |  |  |  |  |
| Unintentional | 1106 | 62.5 (60.2, 64.7) | 951 | 53.9 (51.6, 56.3) | 860 | 50.4 (48.0, 52.8) | 703 | 47.7 (45.1, 50.2) | Reference | 0.12 |
| Intentional | 90 | 52.3 (44.6, 60.0) | 81 | 46.3 (38.7, 54.0) | 76 | 46.1 (38.3, 54.0) | 56 | 39.7 (31.6, 48.3) | 1.13 (0.97, 1.32) |  |
| **Compensable status** |  |  |  |  |  |  |  |  |  |  |
| Non-compensable | 585 | 54.1 (51.1, 57.1) | 504 | 46.6 (43.6, 49.6) | 455 | 43.7 (40.6, 46.7) | 377 | 42.0 (38.8, 45.3) | Reference) | <0.001 |
| Compensable | 610 | 70.4 (67.3, 73.5) | 532 | 61.8 (58.4, 65.0) | 483 | 57.8 (54.4, 61.2) | 387 | 53.3 (49.6, 57.0) | 1.55 (1.38, 1.73) |  |
| **Working prior to injury** |  |  |  |  |  |  |  |  |  |  |
| No | 531 | 68.3 (64.9, 71.6) | 459 | 59.2 (55.6, 62.6) | 436 | 59.0 (55.4, 62.6) | 361 | 60.4 (56.4, 64.4) | Reference | 0.78 |
| Yes | 676 | 57.2 (54.3, 60.0) | 584 | 49.5 (46.6, 52.4) | 508 | 44.2 (41.4, 47.2) | 406 | 39.4 (36.4, 42.4) | 1.01 (0.93, 1.10) |  |
| **Pre-injury disability level** |  |  |  |  |  |  |  |  |  |  |
| None | 864 | 56.7 (54.1, 59.2) | 733 | 48.2 (45.7, 50.8) | 660 | 44.6 (42.0, 47.2) | 543 | 41.8 (39.1, 44.5) | Reference | <0.001 |
| Mild | 184 | 77.0 (71.1, 82.2) | 162 | 68.1 (61.7, 73.9) | 150 | 67.0 (60.4, 73.1) | 119 | 64.7 (57.3, 71.6) | 1.30 (1.19, 1.41) |  |
| Moderate | 103 | 85.1 (77.5, 90.9) | 94 | 77.7 (69.2, 84.8) | 91 | 78.5 (69.9, 85.5) | 68 | 75.6 (65.4, 84.0) | 1.48 (1.34, 1.64) |  |
| Marked/severe | 51 | 75.0 (63.0, 84.7) | 49 | 71.0 (58.8, 81.3) | 42 | 68.9 (55.7, 80.1) | 36 | 69.2 (54.9, 81.3) | 1.37 (1.18, 1.57) |  |
| **Socioeconomic status (IRSAD)** |  |  |  |  |  |  |  |  |  |  |
| 1 – most disadvantaged | 151 | 64.0 (57.5, 70.1) | 141 | 58.8 (52.2, 65.0) | 122 | 52.6 (45.9, 59.2) | 111 | 57.2 (49.9, 64.3) | Reference | 0.62 |
| 2 | 178 | 70.4 (64.3, 75.9) | 146 | 59.1 (52.7, 65.3) | 122 | 53.0 (46.4, 59.6) | 100 | 49.5 (42.4, 56.6) | 1.00 (0.90, 1.12) |  |
| 3 | 206 | 59.0 (53.7, 64.2) | 185 | 52.9 (47.5, 58.2) | 185 | 52.1 (46.8, 57.4) | 138 | 46.2 (40.4, 52.0) | 0.97 (0.87, 1.08) |  |
| 4 | 329 | 59.7 (55.5, 63.8) | 289 | 52.0 (47.7, 56.2) | 269 | 50.6 (46.2, 54.9) | 213 | 45.6 (41.0, 50.3) | 0.94 (0.85, 1.04) |  |
| 5 – most advantaged | 312 | 60.2 (55.9, 64.5) | 260 | 50.8 (46.4, 55.2) | 227 | 46.4 (41.9, 51.0) | 190 | 44.3 (39.5, 49.1) | 0.95 (0.86, 1.05) |  |
| **Nature of injury** |  |  |  |  |  |  |  |  |  |  |
| Isolated head injury | 169 | 62.1 (56.1, 67.9) | 151 | 55.5 (49.4, 61.5) | 128 | 50.8 (44.4, 57.1) | 116 | 54.2 (47.3, 61.0) | Reference | <0.001 |
| Head and other injuries | 293 | 63.4 (58.8, 67.8) | 240 | 54.2 (49.4, 58.9) | 227 | 52.6 (47.7, 57.3) | 170 | 44.7 (39.7, 49.9) | 1.02 (0.92, 1.13) |  |
| Spinal cord injury | 50 | 86.2 (74.6, 93.9) | 51 | 86.4 (75.0, 94.0) | 45 | 77.6 (64.7, 87.5) | 43 | 79.6 (66.5, 89.4) | 1.70 (1.46, 1.98) |  |
| Orthopaedic injuries only | 128 | 67.4 (60.2, 74.0) | 119 | 60.1 (52.9, 67.0) | 100 | 52.6 (45.3, 59.9) | 85 | 51.8 (43.9, 59.7) | 1.12 (1.00, 1.26) |  |
| Chest/abdominal injuries alone | 68 | 38.0 (30.9, 45.5) | 64 | 34.8 (27.9, 42.1) | 60 | 35.5 (28.3, 43.2) | 42 | 29.8 (22.4, 38.1) | 0.70 (0.59, 0.83) |  |
| Chest/abdominal and other injuries | 323 | 63.8 (59.5, 68.0) | 268 | 52.9 (48.4, 57.3) | 254 | 50.2 (45.8, 54.6) | 201 | 46.5 (41.7, 51.4) | 0.98 (0.88, 1.10) |  |
| Other multi-trauma and burns | 176 | 59.9 (54.0, 65.5) | 152 | 51.7 (45.8, 57.5) | 133 | 47.0 (41.1, 53.0) | 112 | 45.3 (39.0, 51.8) | 0.98 (0.87, 1.11) |  |
| **Education** |  |  |  |  |  |  |  |  |  |  |
| University | 174 | 54.6 (48.9, 60.1) | 126 | 40.3 (34.8, 45.9) | 100 | 32.8 (27.5, 38.4) | 85 | 30.5 (25.1, 36.2) | Reference | <0.001 |
| Completed high school | 121 | 54.8 (47.9, 61.4) | 101 | 47.0 (40.2, 53.9) | 92 | 45.1 (38.1, 52.2) | 67 | 37.9 (30.7, 45.4) | 1.17 (1.02, 1.35) |  |
| Diploma or certificate | 348 | 60.6 (56.5, 64.6) | 305 | 54.2 (50.0, 58.3) | 275 | 49.1 (44.9, 53.3) | 235 | 47.3 (42.8, 51.8) | 1.25 (1.12, 1.39) |  |
| Did not complete high school | 447 | 66.3 (62.6, 69.9) | 400 | 57.4 (53.6, 61.1) | 383 | 57.6 (53.7, 61.4) | 303 | 54.8 (50.5, 59.0) | 1.28 (1.15, 1.43) |  |
| **Alcohol/mental health issues** |  |  |  |  |  |  |  |  |  |  |
| No | 873 | 61.5 (58.9, 64.0) | 739 | 52.4 (49.7, 55.0) | 661 | 48.1 (45.4, 50.8) | 555 | 45.8 (43.0, 48.7) | Reference | 0.72 |
| Yes | 309 | 62.3 (57.9, 66.6) | 278 | 56.3 (51.8, 60.7) | 252 | 54.7 (50.0, 59.3) | 196 | 50.7 (45.5, 55.7) | 1.02 (0.93, 1.11) |  |

*Model adjusted for each item presented in this table
