# Supplementary material for: Long-term health status and trajectories of seriously injured patients: A population-based longitudinal study
Source: PLoS Med. 2017 Jul 5;14(7):e1002322. doi: 10.1371/journal.pmed.1002322 (PMC5497942; doi:10.1371/journal.pmed.1002322)
Supplement: S4 Table — (DOCX) [file pmed.1002322.s004.docx]

**S4 Table: Number of patients, prevalence and predictors of reporting some/severe problems on the pain or discomfort item of the EQ-5D-3L - results of multivariable longitudinal analyses**

|  | **6 months**  **N = 1945** | | **12 months**  **N = 1943** | | **24 months**  **N = 1873** | | **36 months**  **N = 1629** | | | **Adjusted relative risk* (95% CI)** | **p-value** |
| --- | --- | --- | --- | --- | --- | --- | --- | --- | --- | --- | --- |
|  | **n** | % problems in each group  (95% CI) | **n** | % problems in each group  (95% CI) | **n** | % problems in each group  (95% CI) | | **n** | % problems in each group  (95% CI) |  |  |
| **Sex** |  |  |  |  |  |  | |  |  |  |  |
| Male | 733 | 52.2 (49.6, 54.8) | 684 | 48.6 (45.9, 51.2) | 600 | 44.2 (41.6, 46.9) | | 556 | 46.6 (43.7, 49.5) | Reference | <0.001 |
| Female | 326 | 60.3 (56.0, 64.4) | 290 | 54.2 (49.9, 58.5) | 280 | 54.3 (49.9, 58.6) | | 256 | 58.7 (53.9, 63.4) | 1.17 (1.09, 1.26) |  |
| **Age** |  |  |  |  |  |  | |  |  |  |  |
| 18-24 years | 128 | 47.6 (41.5, 53.7) | 113 | 43.1 (37.1, 49.4) | 95 | 37.5 (31.6, 43.8) | | 90 | 41.9 (35.2, 48.8) | Reference | <0.001 |
| 25-34 years | 155 | 57.0 (50.9, 62.9) | 144 | 51.6 (45.6, 57.6) | 135 | 50.4 (44.2, 56.5) | | 111 | 47.4 (40.9, 54.0) | 1.24 (1.10, 1.41) |  |
| 35-44 years | 160 | 56.7 (50.7, 62.6) | 154 | 54.2 (48.2, 60.1) | 148 | 54.4 (48.3, 60.4) | | 117 | 49.2 (42.6, 55.7) | 1.30 (1.15, 1.47) |  |
| 45-54 years | 174 | 59.6 (53.7, 65.3) | 156 | 54.0 (48.0, 59.8) | 146 | 51.4 (45.4, 57.4) | | 142 | 54.4 (48.2, 60.6) | 1.32 (1.16, 1.50) |  |
| 55-64 years | 169 | 60.8 (54.8, 66.6) | 153 | 54.6 (48.6, 60.6) | 130 | 47.4 (41.4, 53.5) | | 131 | 51.8 (45.4, 58.1) | 1.29 (1.13, 1.47) |  |
| 65-74 years | 118 | 52.9 (46.1, 59.6) | 106 | 47.1 (40.4, 53.9) | 94 | 42.9 (36.3, 49.8) | | 95 | 50.0 (42.7, 57.3) | 1.16 (1.00, 1.35) |  |
| 75+ years | 155 | 47.1 (41.6, 52.7) | 148 | 45.7 (40.2, 51.3) | 132 | 43.6 (37.9, 49.4) | | 126 | 52.9 (46.4, 59.4) | 1.11 (0.95, 1.30) |  |
| **Charlson comorbidity index** |  |  |  |  |  |  | |  |  |  |  |
| 0 | 699 | 54.5 (51.8, 57.3) | 639 | 49.6 (46.8, 52.3) | 596 | 47.2 (44.4, 50.0) | | 549 | 50.0 (47.0, 53.0) | Reference | 0.71 |
| 1 | 277 | 55.1 (50.6, 59.5) | 255 | 50.8 (46.3, 55.3) | 216 | 46.8 (42.1, 51.4) | | 194 | 48.3 (43.3, 53.3) | 1.02 (0.93, 1.11) |  |
| 2+ | 83 | 51.9 (43.8, 59.8) | 80 | 52.6 (44.4, 60.8) | 68 | 46.0 (37.7, 54.3) | | 69 | 53.9 (44.9, 62.8) | 1.05 (0.93, 1.18) |  |
| **Region** |  |  |  |  |  |  | |  |  |  |  |
| Major cities | 717 | 53.1 (50.4, 55.8) | 667 | 49.9 (47.2, 52.6) | 606 | 47.1 (44.3, 49.9) | | 545 | 48.9 (45.9, 51.9) | Reference | 0.97 |
| Regional or remote | 317 | 58.6 (54.3, 62.8) | 285 | 51.4 (47.1, 55.5) | 256 | 47.8 (43.5, 52.1) | | 249 | 52.6 (48.0, 57.2) | 1.00 (0.88, 1.14) |  |
| **Major trauma service** |  |  |  |  |  |  | |  |  |  |  |
| No | 129 | 45.9 (40.0, 51.9) | 129 | 41.5 (35.9, 47.2) | 124 | 39.9 (34.4, 45.5) | | 104 | 46.0 (39.4, 52.8) | Reference | 0.45 |
| Yes | 930 | 55.9 (53.5, 58.3) | 845 | 51.8 (49.3, 54.2) | 756 | 48.4 (45.9, 50.9) | | 708 | 50.5 (47.8, 53.1) | 1.04 (0.94, 1.15) |  |
| **Cause of injury** |  |  |  |  |  |  | |  |  |  |  |
| Motor vehicle occupant | 317 | 65.4 (60.9, 69.6) | 299 | 61.8 (57.3, 66.1) | 291 | 64.4 (59.8, 68.8) | | 236 | 60.7 (55.6, 65.6) | Reference | 0.26 |
| Motorcyclist | 134 | 64.1 (57.2, 70.6) | 123 | 58.6 (51.6, 65.3) | 121 | 57.6 (50.6, 64.4) | | 109 | 59.9 (52.4, 67.1) | 1.03 (0.94, 1.14) |  |
| Pedal cyclist/pedestrian | 117 | 52.0 (45.3, 58.7) | 111 | 51.2 (44.3, 58.0) | 91 | 41.6 (35.0, 48.4) | | 86 | 43.7 (36.6, 50.9) | 0.91 (0.81, 1.01) |  |
| Low fall (≤ 1m) | 169 | 43.2 (38.3, 48.3) | 160 | 41.7 (36.7, 46.8) | 128 | 35.9 (30.9, 41.1) | | 139 | 49.3 (43.3, 55.3) | 0.99 (0.85, 1.17) |  |
| High fall (>1m) | 145 | 56.2 (49.9, 62.3) | 115 | 44.1 (37.9, 50.3) | 103 | 39.0 (33.1, 45.2) | | 102 | 43.0 (36.6, 49.6) | 1.07 (0.94, 1.21) |  |
| Struck by/collision with | 78 | 47.0 (39.2, 54.9) | 75 | 44.6 (37.0, 52.5) | 61 | 37.0 (29.6, 44.8) | | 69 | 44.8 (36.8, 53.0) | 1.02 (0.86, 1.21) |  |
| Other | 99 | 46.9 (40.0, 53.9) | 91 | 41.6 (35.0, 48.4) | 85 | 41.3 (34.5, 48.3) | | 71 | 37.8 (30.8, 45.1) | 0.94 (0.81, 1.09) |  |
| **Intent** |  |  |  |  |  |  | |  |  |  |  |
| Unintentional | 967 | 55.0 (52.7, 57.4) | 887 | 50.7 (48.3, 53.0) | 801 | 47.3 (44.9, 49.8) | | 746 | 50.7 (48.1, 53.3) | Reference | 0.20 |
| Intentional | 80 | 47.1 (39.4, 54.9) | 77 | 44.5 (37.0, 52.2) | 69 | 42.6 (34.9, 50.6) | | 57 | 40.4 (32.3, 49.0) | 1.11 (0.95, 1.30) |  |
| **Compensable status** |  |  |  |  |  |  | |  |  |  |  |
| Non-compensable | 479 | 44.8 (41.8, 47.8) | 436 | 40.6 (37.6, 43.6) | 365 | 35.3 (32.4, 38.3) | | 366 | 40.9 (37.7, 44.2) | Reference | <0.001 |
| Compensable | 568 | 66.0 (62.7, 69.1) | 529 | 61.9 (58.5, 65.1) | 508 | 61.5 (58.1, 64.8) | | 441 | 60.8 (57.2, 64.4) | 1.52 (1.37, 1.69) |  |
| **Working prior to injury** |  |  |  |  |  |  | |  |  |  |  |
| No | 413 | 53.7 (50.1, 57.3) | 395 | 51.4 (47.8, 55.0) | 357 | 48.9 (45.3, 52.7) | | 326 | 54.8 (50.8, 58.9) | Reference | 0.24 |
| Yes | 646 | 55.0 (52.1, 57.9) | 577 | 49.2 (46.3, 52.1) | 522 | 45.8 (42.8, 48.7) | | 483 | 46.9 (43.8, 49.9) | 0.95 (0.87, 1.03) |  |
| **Pre-injury disability level** |  |  |  |  |  |  | |  |  |  |  |
| None | 795 | 52.5 (50.0, 55.1) | 725 | 48.0 (45.4, 50.5) | 659 | 44.8 (42.3, 47.4) | | 610 | 46.9 (44.2, 49.7) | Reference | <0.001 |
| Mild | 138 | 58.5 (51.9, 64.8) | 137 | 57.8 (51.2, 64.2) | 122 | 55.2 (48.4, 61.9) | | 115 | 63.2 (55.7, 70.2) | 1.28 (1.17, 1.40) |  |
| Moderate | 80 | 66.7 (57.5, 75.0) | 69 | 59.0 (49.5, 68.0) | 61 | 53.5 (43.9, 62.9) | | 53 | 59.6 (48.6, 69.8) | 1.32 (1.16, 1.51) |  |
| Marked/severe | 43 | 64.2 (51.5, 75.5) | 36 | 52.9 (40.4, 65.2) | 35 | 58.3 (44.9, 70.9) | | 31 | 59.6 (45.1, 73.0) | 1.28 (1.08, 1.51) |  |
| **Socioeconomic status** |  |  |  |  |  |  | |  |  |  |  |
| 1 – most disadvantaged | 152 | 65.0 (58.5, 71.1) | 144 | 60.0 (53.5, 66.2) | 133 | 57.3 (50.7, 63.8) | | 112 | 57.7 (50.4, 64.8) | Reference | 0.001 |
| 2 | 153 | 61.0 (54.6, 67.0) | 138 | 56.3 (49.9, 62.6) | 108 | 47.4 (40.7, 54.1) | | 102 | 50.5 (43.4, 57.6) | 0.90 (0.80, 1.00) |  |
| 3 | 208 | 60.3 (54.9, 65.5) | 171 | 49.3 (43.9, 54.7) | 177 | 50.0 (44.7, 55.3) | | 162 | 54.4 (48.5, 60.1) | 0.90 (0.81, 0.99) |  |
| 4 | 280 | 51.2 (46.9, 55.5) | 269 | 48.7 (44.5, 53.0) | 243 | 46.1 (41.8, 50.5) | | 233 | 49.9 (45.3, 54.5) | 0.84 (0.77, 0.93) |  |
| 5 – most advantaged | 241 | 46.9 (42.5, 51.3) | 230 | 45.4 (41.0, 49.8) | 201 | 41.7 (37.3, 46.2) | | 185 | 43.3 (38.6, 48.2) | 0.81 (0.73, 0.90) |  |
| **Nature of injury** |  |  |  |  |  |  | |  |  |  |  |
| Isolated head injury | 98 | 36.3 (30.6, 42.3) | 95 | 35.5 (29.7, 41.5) | 83 | 33.2 (27.4, 39.4) | | 87 | 40.9 (34.2, 47.8) | Reference | <0.001 |
| Head and other injuries | 244 | 53.5 (48.8, 58.2) | 211 | 48.2 (43.4, 53.0) | 191 | 44.7 (39.9, 49.6) | | 175 | 46.1 (41.0, 51.2) | 1.21 (1.06, 1.39) |  |
| Spinal cord injury | 44 | 77.2 (64.2, 87.3) | 42 | 71.2 (57.9, 82.2) | 39 | 67.2 (53.7, 79.0) | | 43 | 79.6 (66.5, 89.4) | 1.92 (1.62, 2.27) |  |
| Orthopaedic injuries only | 123 | 64.7 (57.5, 71.5) | 115 | 58.1 (50.9, 65.0) | 105 | 55.9 (48.4, 63.1) | | 99 | 60.7 (52.8, 68.3) | 1.50 (1.32, 1.74) |  |
| Chest/abdominal injuries alone | 68 | 38.4 (31.2, 46.0) | 73 | 39.9 (32.7, 47.4) | 57 | 33.7 (26.6, 41.4) | | 43 | 30.5 (23.0, 38.8) | 0.95 (0.80, 1.15) |  |
| Chest/abdominal and other injuries | 318 | 63.1 (58.7, 67.3) | 290 | 57.3 (52.9, 61.7) | 275 | 54.9 (50.4, 59.3) | | 239 | 55.5 (50.6, 60.2) | 1.35 (1.20, 1.57) |  |
| Other multi-trauma and burns | 164 | 56.4 (50.4, 62.1) | 148 | 50.9 (45.0, 56.7) | 130 | 46.4 (40.5, 52.5) | | 126 | 51.0 (44.6, 57.4) | 1.30 (1.12, 1.49) |  |
| **Education** |  |  |  |  |  |  | |  |  |  |  |
| University | 150 | 47.2 (41.6, 52.8) | 130 | 41.9 (36.4, 47.6) | 104 | 34.3 (29.0, 40.0) | | 99 | 35.5 (29.9, 41.4) | Reference | <0.001 |
| Completed high school | 108 | 48.9 (42.1, 55.7) | 106 | 49.3 (42.4, 56.2) | 88 | 43.6 (36.6, 50.7) | | 87 | 49.4 (41.9, 57.1) | 1.20 (1.05, 1.37) |  |
| Diploma or certificate | 329 | 57.6 (53.4, 61.7) | 290 | 51.8 (47.6. 56.0) | 276 | 49.6 (45.3, 53.8) | | 265 | 53.3 (48.8, 57.8) | 1.27 (1.14, 1.41) |  |
| Did not complete high school | 381 | 57.4 (53.5, 61.2) | 360 | 52.1 (48.3, 55.9) | 346 | 52.6 (48.7, 56.5) | | 587 | 52.1 (47.8, 56.3) | 1.26 (1.13, 1.41) |  |
| **Alcohol/mental health issues** |  |  |  |  |  |  | |  |  |  |  |
| No | 764 | 54.3 (51.6, 56.9) | 706 | 50.4 (47.7, 53.0) | 639 | 46.9 (44.2, 49.6) | | 604 | 50.0 (47.1, 52.8) | Reference | 0.40 |
| Yes | 270 | 54.9 (50.4, 59.3) | 243 | 49.7 (45.2, 54.2) | 212 | 46.6 (41.9, 51.3) | | 185 | 47.9 (42.8, 53.0) | 0.96 (0.87, 1.06) |  |

*Model adjusted for each item presented in this table
