# Supplementary material for: Long-term health status and trajectories of seriously injured patients: A population-based longitudinal study
Source: PLoS Med. 2017 Jul 5;14(7):e1002322. doi: 10.1371/journal.pmed.1002322 (PMC5497942; doi:10.1371/journal.pmed.1002322)
Supplement: S5 Table — (DOCX) [file pmed.1002322.s005.docx]

**S5 Table: Number of patients, prevalence and predictors of reporting some/severe problems on the anxiety or depression item of the EQ-5D-3L - results of multivariable longitudinal analyses**

|  | **6 months**  **N = 1939** | | **12 months**  **N = 1938** | | **24 months**  **N = 1875** | | **36 months**  **N = 1628** | | **Adjusted relative risk* (95% CI)** | **p-value** |
| --- | --- | --- | --- | --- | --- | --- | --- | --- | --- | --- |
|  | **n** | % problems in each group  (95% CI) | **n** | % problems in each group  (95% CI) | **n** | % problems in each group  (95% CI) | **n** | % problems in each group  (95% CI) |  |  |
| **Sex** |  |  |  |  |  |  |  |  |  |  |
| Male | 605 | 43.2 (40.5, 45.8) | 568 | 40.5 (37.9, 43.1) | 502 | 37.0 (34.4, 39.6) | 448 | 37.6 (34.8, 40.4) | Reference | <0.001 |
| Female | 264 | 49.2 (44.9, 53.5) | 265 | 49.5 (45.2, 53.9) | 245 | 47.4 (43.0, 51.8) | 216 | 49.5 (44.8, 54.3) | 1.20 (1.10, 1.31) |  |
| **Age** |  |  |  |  |  |  |  |  |  |  |
| 18-24 years | 111 | 41.3 (35.3, 47.4) | 97 | 37.0 (31.2, 43.2) | 90 | 35.2 (29.3, 41.3) | 88 | 40.9 (34.3, 47.8) | Reference | <0.001 |
| 25-34 years | 148 | 54.2 (48.1, 60.2) | 139 | 49.6 (43.6, 55.7) | 114 | 42.7 (36.7, 48.9) | 101 | 43.2 (36.7, 49.8) | 1.21 (1.05, 1.39) |  |
| 35-44 years | 131 | 46.1 (40.2, 52.1) | 129 | 45.4 (39.5, 51.4) | 127 | 46.5 (40.5, 52.6) | 98 | 41.2 (34.9, 47.7) | 1.19 (1.03, 1.39) |  |
| 45-54 years | 146 | 49.8 (43.9, 55.7) | 133 | 45.7 (39.9, 51.6) | 125 | 44.0 (38.2, 50.0) | 113 | 43.5 (37.3, 49.7) | 1.20 (1.04, 1.39) |  |
| 55-64 years | 126 | 46.0 (40.0, 52.1) | 133 | 47.2 (41.2, 53.2) | 108 | 39.4 (33.6, 45.5) | 96 | 37.8 (31.8, 44.1) | 1.11 (0.95, 1.29) |  |
| 65-74 years | 92 | 41.1 (34.6, 47.8) | 77 | 34.5 (28.3, 41.2) | 70 | 32.1 (26.0, 38.7) | 73 | 38.4 (31.5, 45.7) | 0.88 (0.73, 1.05) |  |
| 75+ years | 115 | 35.7 (30.5, 41.2) | 125 | 39.6 (34.1, 45.2) | 113 | 37.3 (31.8, 43.0) | 95 | 40.1 (33.8, 46.6) | 0.76 (0.64, 0.91) |  |
| **Charlson comorbidity index** |  |  |  |  |  |  |  |  |  |  |
| 0 | 525 | 41.0 (38.3, 43.7) | 502 | 39.0 (36.3, 41.7) | 454 | 36.1 (33.4, 38.8) | 423 | 38.5 (35.6, 41.5) | Reference | 0.30 |
| 1 | 266 | 53.3 (48.8, 57.8) | 253 | 50.5 (46.0, 55.0) | 222 | 47.2 (42.6, 51.9) | 188 | 46.9 (41.9, 51.9) | 1.05 (0.94, 1.18) |  |
| 2+ | 78 | 49.1 (41.1, 57.1) | 78 | 52.4 (44.0, 60.6) | 71 | 48.3 (40.0, 56.7) | 53 | 41.1 (32.5, 50.1) | 1.11 (0.97, 1.27) |  |
| **Region** |  |  |  |  |  |  |  |  |  |  |
| Major cities | 608 | 45.2 (42.5, 47.9) | 585 | 43.9 (41.2, 46.6) | 521 | 40.5 (37.8, 43.2) | 464 | 41.7 (38.7, 44.6) | Reference | 0.27 |
| Regional or remote | 234 | 43.3 (39.1, 47.6) | 229 | 41.3 (37.1, 45.5) | 204 | 37.9 (33.7, 42.1) | 185 | 39.1 (34.7, 43.7) | 0.90 (0.76, 1.08) |  |
| **Major trauma service** |  |  |  |  |  |  |  |  |  |  |
| No | 117 | 41.8 (35.9, 47.8) | 109 | 35.5 (30.2, 41.1) | 114 | 36.8 (31.4, 42.4) | 85 | 37.6 (31.3, 44.3) | Reference | 0.61 |
| Yes | 752 | 45.3 (42.9, 47.8) | 724 | 44.4 (42.0, 46.8) | 633 | 40.5 (38.0, 42.9) | 579 | 41.3 (38.7, 43.9) | 1.03 (0.92, 1.16) |  |
| **Cause of injury** |  |  |  |  |  |  |  |  |  |  |
| Motor vehicle occupant | 255 | 52.7 (48.1, 57.2) | 253 | 52.5 (47.9, 57.0) | 232 | 51.2 (46.5, 55.9) | 194 | 50.0 (44.9, 55.1) | Reference | 0.05 |
| Motorcyclist | 89 | 43.0 (36.2, 50.0) | 80 | 38.3 (31.7, 45.2) | 79 | 37.8 (31.2, 44.7) | 67 | 36.8 (29.8, 44.3) | 0.87 (0.75, 1.01) |  |
| Pedal cyclist/pedestrian | 85 | 38.3 (31.9, 45.0) | 86 | 39.8 (33.2, 46.7) | 81 | 36.8 (30.4, 43.6) | 74 | 37.4 (30.6, 44.5) | 0.88 (0.77, 1.01) |  |
| Low fall (≤ 1m) | 168 | 43.0 (38.0, 48.0) | 157 | 40.9 (35.9, 46.0) | 130 | 36.6 (31.6, 41.9) | 115 | 40.9 (35.1, 46.9) | 1.10 (0.91, 1.34) |  |
| High fall (>1m) | 93 | 36.2 (30.3, 42.4) | 74 | 28.4 (23.0, 34.2) | 65 | 24.5 (19.5, 30.2) | 69 | 29.1 (23.4, 35.3) | 0.90 (0.75, 1.08) |  |
| Struck by/collision with | 88 | 53.3 (45.4, 61.1) | 83 | 49.4 (41.6, 57.2) | 71 | 42.8 (35.1, 50.7) | 69 | 44.8 (36.8, 53.0) | 1.09 (0.89, 1.33) |  |
| Other | 91 | 42.7 (36.0, 49.7) | 100 | 45.9 (39.1, 52.7) | 89 | 43.0 (36.2, 50.0) | 76 | 40.4 (33.3, 47.8) | 1.06 (0.88, 1.26) |  |
| **Intent** |  |  |  |  |  |  |  |  |  |  |
| Unintentional | 749 | 42.8 (40.5, 45.2) | 721 | 41.3 (39.0, 43.6) | 644 | 38.0 (35.7, 40.4) | 577 | 39.2 (36.7, 41.8) | Reference | <0.001 |
| Intentional | 109 | 63.7 (56.1, 70.9) | 102 | 59.0 (51.2, 66.4) | 94 | 57.7 (49.7, 65.4) | 76 | 53.9 (45.3, 62.3) | 1.40 (1.20, 1.63) |  |
| **Compensable status** |  |  |  |  |  |  |  |  |  |  |
| Non-compensable | 428 | 40.0 (37.0, 43.0) | 403 | 37.6 (34.7, 40.6) | 340 | 32.9 (30.0, 35.8) | 315 | 35.2 (32.1, 38.5) | Reference | <0.001 |
| Compensable | 433 | 50.7 (47.3, 54.1) | 420 | 49.2 (45.8, 52.6) | 401 | 48.5 (45.0, 52.0) | 345 | 47.6 (43.9, 51.3) | 1.58 (1.37, 1.81) |  |
| **Working prior to injury** |  |  |  |  |  |  |  |  |  |  |
| No | 383 | 50.3 (46.7, 53.9) | 372 | 48.8 (45.2, 52.4) | 343 | 47.0 (43.4, 50.7) | 287 | 48.2 (44.2, 52.4) | Reference | <0.001 |
| Yes | 485 | 41.3 (38.4, 44.2) | 459 | 39.1 (36.3, 42.0) | 403 | 35.3 (32.5, 38.1) | 373 | 36.3 (33.3, 39.2) | 0.80 (0.72, 0.88) |  |
| **Pre-injury disability level** |  |  |  |  |  |  |  |  |  |  |
| None | 599 | 39.7 (37.2, 42.2) | 582 | 38.6 (36.1, 41.1) | 523 | 35.5 (33.1, 38.0) | 468 | 36.1 (33.4, 38.7) | Reference | <0.001 |
| Mild | 136 | 57.6 (51.0, 64.0) | 121 | 51.5 (44.9, 58.0) | 110 | 50.5 (43.6, 57.3) | 102 | 56.0 (48.5, 63.4) | 1.44 (1.29, 1.61) |  |
| Moderate | 85 | 71.4 (62.4, 79.3) | 74 | 62.2 (52.8, 70.9) | 66 | 56.9 (47.4, 66.1) | 57 | 63.3 (52.5, 73.2) | 1.50 (1.30, 1.72) |  |
| Marked/severe | 44 | 66.7 (54.0, 77.8) | 47 | 71.2 (58.7, 81.7) | 42 | 68.9 (55.7, 80.1) | 34 | 65.4 (50.9, 78.0) | 1.58 (1.37, 1.82) |  |
| **Socioeconomic status** |  |  |  |  |  |  |  |  |  |  |
| 1 – most disadvantaged | 130 | 56.3 (49.6, 62.8) | 124 | 52.1 (45.6, 58.6) | 102 | 44.4 (37.8, 51.0) | 98 | 50.5 (43.3, 57.8) | Reference | 0.04 |
| 2 | 120 | 47.8 (41.5, 54.2) | 110 | 44.5 (38.2, 51.0) | 91 | 39.6 (33.2, 46.2) | 74 | 36.6 (30.0, 43.7) | 0.87 (0.75, 1.00) |  |
| 3 | 155 | 44.5 (39.2, 49.9) | 159 | 45.8 (40.5, 51.2) | 151 | 42.8 (37.6, 48.1) | 132 | 44.3 (38.6, 50.1) | 0.89 (0.78, 1.00) |  |
| 4 | 232 | 42.7 (38.5, 47.0) | 232 | 42.1 (37.9, 46.4) | 207 | 39.1 (34.9, 43.4) | 196 | 42.1 (37.5, 46.7) | 0.86 (0.76, 0.97) |  |
| 5 – most advantaged | 205 | 40.0 (35.7, 44.3) | 189 | 37.5 (33.3, 41.9) | 174 | 36.0 (31.7, 40.4) | 149 | 34.9 (30.4, 39.6) | 0.83 (0.74, 0.95) |  |
| **Nature of injury** |  |  |  |  |  |  |  |  |  |  |
| Isolated head injury | 121 | 45.2 (39.1, 51.3) | 119 | 44.4 (38.6, 50.6) | 103 | 41.2 (35.0, 47.6) | 94 | 44.1 (37.4, 51.1) | Reference | 0.07 |
| Head and other injuries | 218 | 48.0 (43.3, 52.7) | 197 | 44.9 (40.2, 49.7) | 178 | 41.6 (36.9, 46.4) | 170 | 44.9 (39.8, 50.0) | 0.99 (0.86, 1.13) |  |
| Spinal cord injury | 25 | 43.1 (30.2, 56.8) | 28 | 47.5 (34.3, 60.9) | 21 | 36.2 (24.0, 49.9) | 28 | 51.9 (37.8, 65.7) | 1.17 (0.93, 1.46) |  |
| Orthopaedic injuries only | 82 | 43.4 (36.2, 50.8) | 83 | 42.1 (35.1, 49.4) | 61 | 32.5 (25.8, 39.6) | 52 | 31.9 (24.8, 39.6) | 0.88 (0.75, 1.04) |  |
| Chest/abdominal injuries alone | 73 | 41.0 (33.7, 48.6) | 66 | 36.3 (29.3, 43.7) | 55 | 33.1 (26.0, 40.8) | 43 | 30.5 (23.0, 38.8) | 0.82 (0.68, 0.97) |  |
| Chest/abdominal and other injuries | 215 | 43.0 (38.6, 47.5) | 220 | 43.7 (39.3, 48.1) | 205 | 40.8 (36.5, 45.3) | 171 | 39.7 (35.0, 44.5) | 0.96 (0.83, 1.11) |  |
| Other multi-trauma and burns | 135 | 46.2 (40.4, 52.1) | 120 | 41.5 (35.8, 47.4) | 124 | 43.8 (38.0, 49.8) | 106 | 42.9 (36.7, 49.3) | 0.95 (0.82, 1.10) |  |
| **Education** |  |  |  |  |  |  |  |  |  |  |
| University | 111 | 35.2 (30.0, 40.8) | 92 | 29.7 (24.6, 35.1) | 77 | 25.4 (20.6, 30.7) | 78 | 28.0 (22.8, 33.6) | Reference | 0.002 |
| Completed high school | 93 | 42.3 (35.7, 49.1) | 96 | 44.9 (38.1, 51.8) | 79 | 39.1 (32.3, 46.2) | 73 | 41.5 (34.1, 49.1) | 1.28 (1.09, 1.51) |  |
| Diploma or certificate | 248 | 43.4 (39.3, 47.5) | 226 | 40.4 (36.3, 44.6) | 229 | 41.0 (36.9, 45.2) | 205 | 41.4 (37.0, 45.9) | 1.29 (1.12, 1.48) |  |
| Did not complete high school | 321 | 48.1 (44.3, 52.0) | 324 | 46.8 (43.1, 50.6) | 289 | 43.9 (40.0, 47.7) | 242 | 43.8 (39.6, 48.0) | 1.32 (1.14, 1.53) |  |
| **Alcohol/mental health issues** |  |  |  |  |  |  |  |  |  |  |
| No | 571 | 40.7 (38.1, 43.4) | 541 | 38.8 (36.2, 41.4) | 486 | 35.7 (33.2, 38.3) | 454 | 37.6 (34.9, 40.4) | Reference | 0.04 |
| Yes | 281 | 57.1 (52.6, 61.5) | 270 | 55.0 (50.5, 59.5) | 240 | 52.2 (47.5, 56.8) | 193 | 49.9 (44.8, 55.0) | 1.13 (1.01, 1.26) |  |

*Model adjusted for each item presented in this table
